# Anurans from the Lower Cretaceous Jehol Group of Western Liaoning, China

**DOI:** 10.1371/journal.pone.0069723

**Published:** 2013-07-26

**Authors:** Liping Dong, Zbyněk Roček, Yuan Wang, Marc E H. Jones

**Affiliations:** 1 Key Laboratory of Vertebrate Evolution and Human Origin of Chinese Academy of Sciences, Institute of Vertebrate Paleontology and Paleoanthropology, Chinese Academy of Sciences, Beijing, China; 2 Institute of Geology, Department of Palaeobiology, Academy of Sciences of the Czech Republic, Prague, Czech Republic; 3 Research Department of Cell and Developmental Biology, University College London, London, United Kingdom; Raymond M. Alf Museum of Paleontology, United States of America

## Abstract

**Background:**

To date, the Lower Cretaceous Jehol Group of western Liaoning, China has yielded five monotypic genera of anurans, including *Liaobatrachus grabaui*, *Callobatrachus sanyanensis*, *Mesophryne beipiaoensis*, *Dalianbatrachus mengi*, and *Yizhoubatrachus macilentus*. However, the validity and distinctness of these taxa have been questioned.

**Methodology/Principal Finding:**

We provide a comprehensive analysis of the Jehol frogs that includes a re-examination of the published taxa as well as an examination of a number of new specimens that have been collected over the past 10 years. The results show that the five previously named taxa can be referred to three species of one genus–*Liaobatrachus grabaui*, *L*. *beipiaoensis* comb. nov. and *L*. *macilentus* comb. nov.. The diagnosis of *Liaobatrachus* is revised, and a new diagnosis is provided for each species of this genus. We also establish *Liaobatrachus zhaoi* sp. nov., on the basis of a dozen well-preserved specimens from a new locality. This taxon is distinguished by a unique combination of characteristics, including relatively long hind limbs, a rounded rather than triangular acetabulum, and a gradually-tapering cultriform process of the parasphenoid. In addition, an unnamed frog from a higher horizon, which has narrow sacral diapophyses and particularly long legs, is different from *Liaobatrachus* and represents another form of anuran in the Jehol Biota.

**Conclusion/Significance:**

Comparisons with other Mesozoic and extant anurans and the primary phylogenetic analysis both suggest that *Liaobatrachus* is a member of the anuran crown-group and forms a polytomy with leiopelmatids (*Ascaphus* and *Leiopelma*) and the remaining crown-group anurans (Lalagobatrachia).

## Introduction

The Jehol Biota represents an Early Cretaceous lacustrine terrestrial ecosystem with a high degree of diversity and endemism [1], [2]. It contains a variety of Mesozoic taxa including the early bird *Confuciusornis* and its relatives [3], the feathered tyrannosauroid *Yutyrannus huali* [4], and the early eutherian *Acristatherium yanensis* [5]. The Jehol Biota is best known from the Jehol Group, which is exposed in western Liaoning, northern Hebei and southeastern Inner Mongolia. The Jehol Group consists of, in ascending order, the Dabeigou Formation, the Yixian Formation and the Jiufotang Formation. Its age has been estimated as 129.7±0.5–122.1±0.3 Ma [6] or 131–120 Ma [2] by different recent studies, indicating that the biota lasted about 7–9 Ma (early Barremian to early Aptian).

Amphibians are an important component of the Jehol Biota, with 4 urodele [7] and 5 anuran taxa having been formally reported [8]–[12]. The anurans include *Liaobatrachus grabaui* Ji and Ji 1998 [8], *Callobatrachus sanyanensis* Wang and Gao 1999 [9], *Mesophryne beipiaoensis* Gao and Wang 2001 [10], *Dalianbatrachus mengi* Gao and Liu 2004 [11], and *Yizhoubatrachus macilentus* Gao and Chen 2004 [12]. Because each taxon was based on a single specimen, some anatomical characters were ambiguous or even incorrectly interpreted. For example, *Liaobatrachus grabaui* was reported to have no ribs [8], the centra of *Mesophryne beipiaoensis* were reported to be procoelous [10], and *Dalianbatrachus mengi* was considered to have fused frontoparietals [11]. Some revisions have been made [13], but many problems and uncertainties remain unresolved. Based on new fossil discoveries and a re-examination of all reported taxa, this paper provides the first comprehensive study of the Jehol anurans, including revisions of established taxa and erection of a new taxon.

## Materials and Methods

### Institutional Abbreviations

CYH, Chaoyang Bird Fossil National Geopark, Chaoyang, Liaoning; DNM D, Dalian Natural History Museum, Dalian, Liaoning; GMV, Geological Museum of China, Beijing; IVPP V, Institute of Vertebrate Paleontology and Paleoanthropology, Chinese Academy of Sciences, Beijing; LPM, Liaoning Paleontology Museum, Shenyang, Liaoning; MV, Nanjing Institute of Geology and Palaeontology, Chinese Academy of Sciences, Nanjing, Jiangsu; ZMNH M, Zhejiang Museum of Natural History, Hangzhou, Zhejiang.

### Localities and Materials

**Figure 1:**
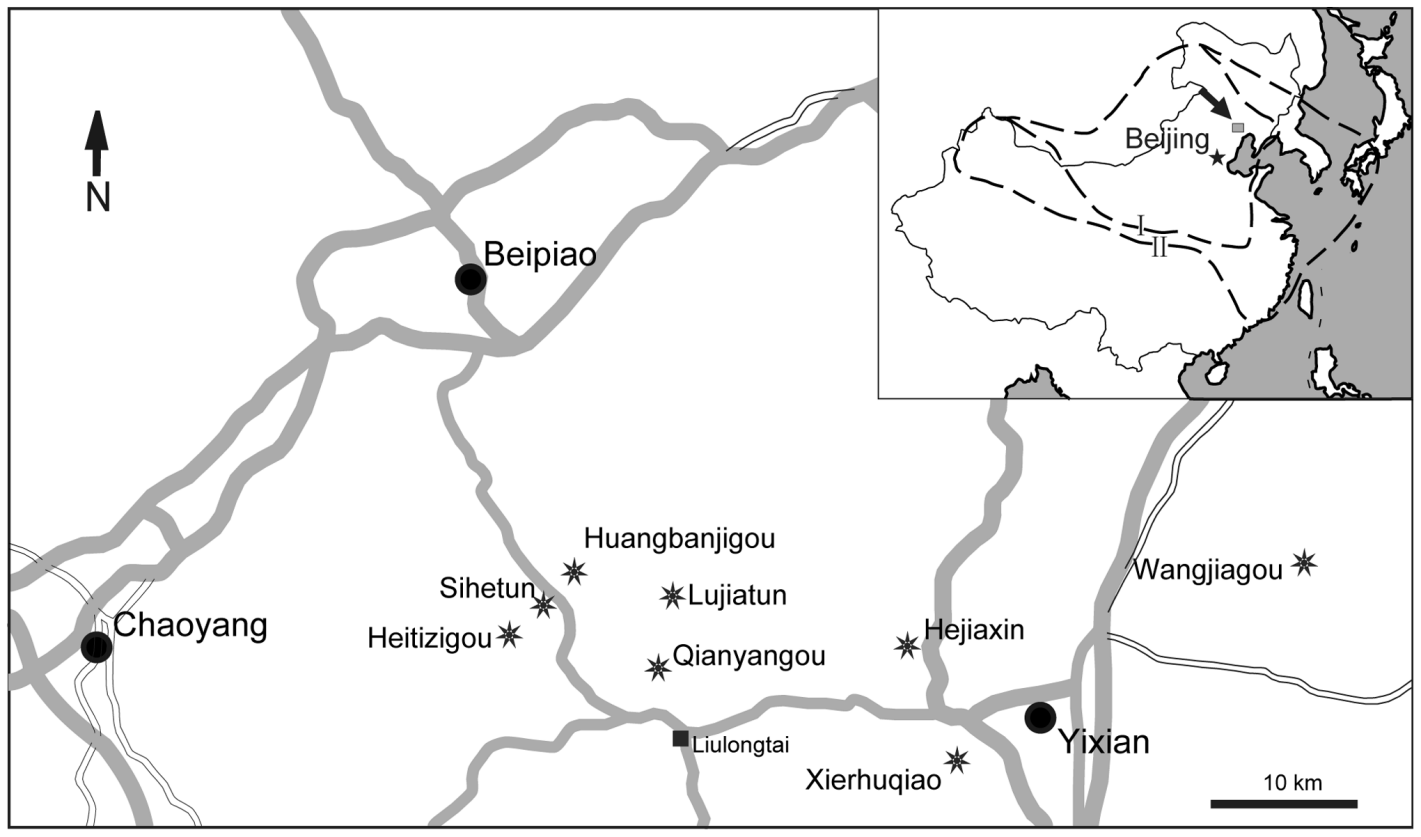
Localities for Early Cretaceous fossil anurans from Western Liaoning, China described in this paper. The location of the Beipiao-Yixian area is indicated on the small-scale map (modified from Zhou et al., 2003[1]) by a black arrow. Each locality is indicated by a star on the large-scale map.

**Figure 2:**
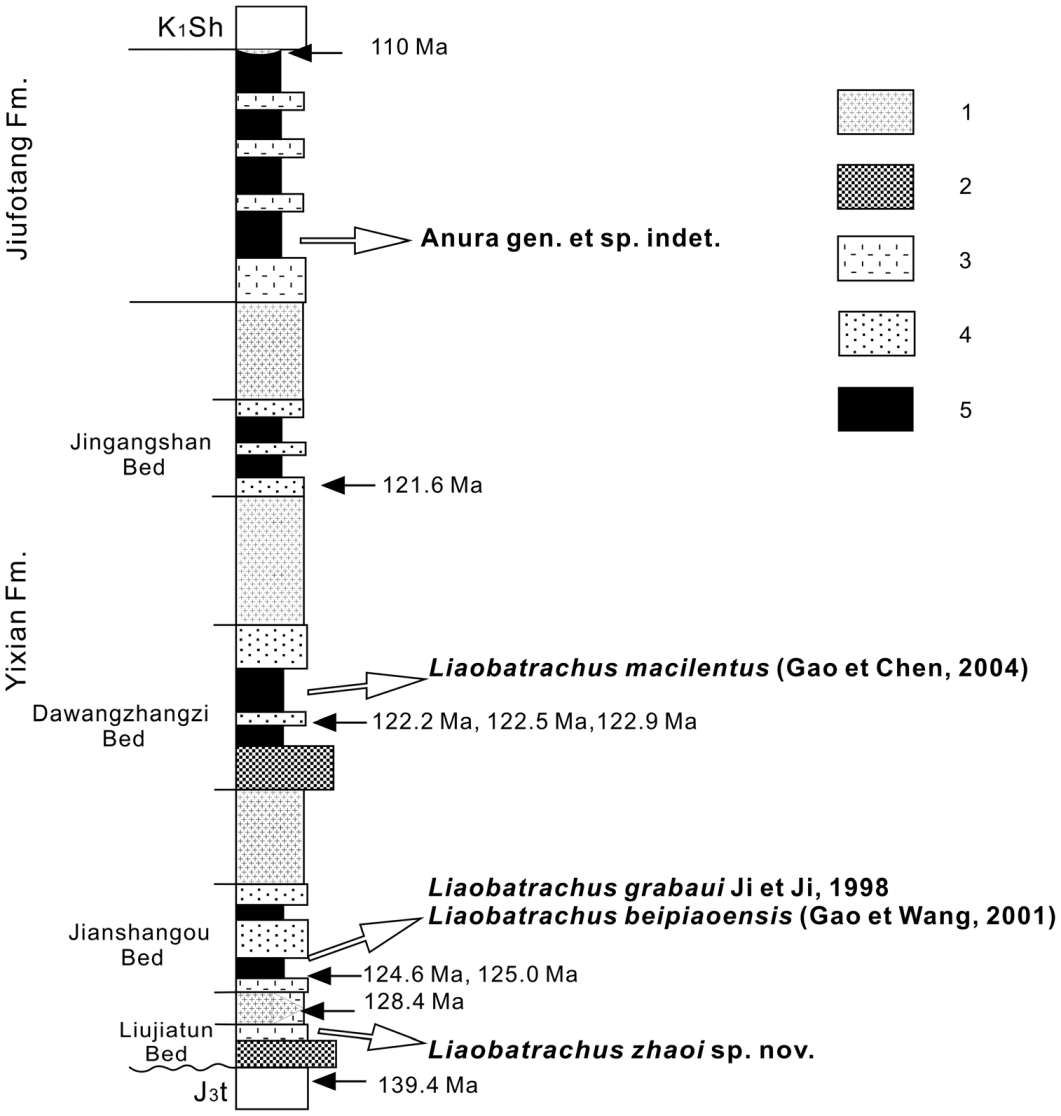
Stratigraphic distribution of Jehol anurans described in this paper. Based on stratigraphic scheme in Chang et al. (2003) [14]. 1, basalt and andesite with volcanic breccia (lava); 2, conglomerate with volcanic breccia; 3, sandstone and conglomerate; 4, tuffaceous sandstone and tuff; 5, shale and tuff.

We examined 25 specimens from eight fossil localities in the area around Yixian, Beipiao and Chaoyang in western Liaoning (Fig. 1). The anuran-bearing horizons are the Lujiatun, Jianshangou and Dawangzhangzi Beds of the Yixian Formation, and the Jiufotang Formation (Fig. 2) [14]. There are not anurans currently known from the Dabeigou Formation. The localities and their anuran fossil content are as follows (also see Table 1):

**Table 1:** List of anuran specimens examined in this paper (arranged according to geological age).

Specimen	Age	Horizon	Locality
IVPP V13245	Barremian	Lujiatun Bed	Lujiatun
IVPP V13236	Barremian	Lujiatun Bed	Qianyangou
IVPP V13239	Barremian	Lujiatun Bed	Qianyangou
IVPP V13380	Barremian	Lujiatun Bed	Qianyangou
IVPP V14203	Barremian	Lujiatun Bed	Qianyangou
IVPP V14269	Barremian	Lujiatun Bed	Qianyangou
IVPP V14270	Barremian	Lujiatun Bed	Qianyangou
IVPP V14979.1	Barremian	Lujiatun Bed	Qianyangou
IVPP V14979.2	Barremian	Lujiatun Bed	Qianyangou
*IVPP V14979.3	Barremian	Lujiatun Bed	Qianyangou
*IVPP V14979.4	Barremian	Lujiatun Bed	Qianyangou
IVPP V13238	Barremian	Lujiatun Bed	Qianyangou
IVPP V13379	Barremian	Lujiatun Bed	Qianyangou
IVPP V14068	Barremian	Lujiatun Bed	Qianyangou
GMV2126	Barremian/Aptian	Jianshangou Bed	Sihetun
IVPP V11525	Barremian/Aptian	Jianshangou Bed	Sihetun
IVPP V12717	Barremian/Aptian	Jianshangou Bed	Sihetun
MV 77	Barremian/Aptian	Jianshangou Bed	Sihetun
CYH 004	Barremian/Aptian	Jianshangou Bed	Sihetun
LPM 0030	Barremian/Aptian	Jianshangou Bed	Heitizigou
DNM D2166/7	Barremian/Aptian	Jianshangou Bed	Huangbanjigou
ZMNH M8621	Aptian	Dawangzhangzi Bed	Hejiaxin
IVPP V12510	Aptian	Dawangzhangzi Bed	Hejiaxin
*IVPP V12541	Aptian	Dawangzhangzi Bed	WangJiagou
*IVPP V13235	Aptian	Jiufotang Forma tion	Xierhuqiao

* juvenile individual.

1. Lujiatun Locality: near Lujiatun Village, 25 km northwest of Beipiao City, Liaoning Province; Lujiatun Bed, Yixian Formation; Barremian. Specimen IVPP V13245 (a three-dimensionally preserved incomplete skeleton) was excavated here.
2. Qianyangou Locality: about 1.5 km west of Qianyangou Village, Shangyuan Town, Beipiao City, Liaoning Province; Lujiatun Bed, Yixian Formation; Barremian. All of the specimens from this locality are three-dimensionally preserved. They include IVPP V13236 (a partial skeleton with part of the skull and most of the postcranial bones preserved), IVPP V13239 (a partial skeleton lacking the urostyle, ilia, and hind legs), IVPP V13380 (a partial skeleton with the skull and pectoral girdle preserved), IVPP V14203 (a nearly complete skeleton missing only some limb bones), IVPP V14269 (the smaller of two frogs preserved on one slab, with the upper and lower jaws and most of the postcranial bones preserved), IVPP V14270 (the larger on the slab that also bears specimen IVPP V14269, a partial skeleton with part of the skull, part of the appendicular skeleton and nearly all of the vertebral column preserved), IVPP V13238 (a partial skeleton with part of the vertebral column, the pelvis and the hind limbs preserved), IVPP V13379 (a partial skeleton with most of the cranial bones missing), and IVPP V14608 (a partial skeleton consisting of the posterior portion of the vertebral column, the pelvis and the hind limbs). Specimen IVPP V14979, which represents several frogs preserved on two associated slabs, was also recovered from this locality. Some of the skeletons are nearly complete, with only a few bones disarticulated and displaced. The first slab bears two adult skeletons (IVPP V14979.1 and IVPP V14979.2) and two juvenile skeletons (IVPP V14979.3 and IVPP V14979.4), whereas the second slab (not considered in the paper as they are the young individuals of IVPP V14979.1) bears two frogs: IVPP V14979.5 and IVPP V14979.6 [15].
3. Sihetun Locality: 1.5 km southwest of Sihetun Cunminzu (a “Cunminzu” is a subdivision of a village), Chaomidianzi Village, Shangyuan Town, Beipiao City, Liaoning Province; Jianshangou Bed, Yixian Formation; Barremian/Aptian. Specimens found here include GMV2126 (see Ji and Ji, 1998 [8]), IVPP V11525 (see Wang and Gao, 1999 [9]), MV 77 (a nearly complete skeleton with a slightly displaced pelvis), IVPP V12717 (a nearly complete skeleton with part of the vertebral column displaced and the limbs not preserved), and CYH 004 (a nearly complete skeleton).
4. Heitizigou Locality: 0.5 km east of Libalanggou Village, Zhangjiying Township, Beipiao City, Liaoning Province; Jianshangou Bed, Yixian Formation; Barremian/Aptian. Specimen LPM 0030 (see Gao and Wang, 2001 [10]) was found here.
5. Huangbanjigou Locality: near Huangbanjigou Village, Shangyuan Town, Beipiao City, Liaoning Province, 3 km northeast of the Sihetun Locality; Jianshangou Bed, Yixian Formation; Barremian/Aptian. Specimen DNM D2166/7 (see Gao and Liu, 2004 [11]) was found here.
6. Hejiaxin Locality: near Hejiaxin Village 15 km west of the Yixian County, Jinzhou City, Liaoning Province; Dawangzhangzi Bed, Yixian Formation; Aptian. Specimens ZMNH M8621 (see Gao and Chen, 2004 [12]) and IVPP V12510 (a nearly complete skeleton) were found here.
7. Wangjiagou Locality: about 20 km northeast of Yixian, Jinzhou City, Liaoning Province; Dawangzhangzi Bed, Yixian Formation; Aptian. Specimen IVPP V12541 (a nearly complete skeleton with disarticulated and displaced cranial bones) was found here.
8. Xierhuqiao Locality: 6 km southwest of Yixian, Jinzhou City, Liaoning Province; Jiufotang Formation; Aptian. Specimen IVPP V13235 (see Wang et al., 2007 [16]) was recovered here.

All of the specimens mentioned above are owned by and deposited in museums or research institutes, and access to them was granted through proper official channels.

### Nomenclatural Acts

The electronic edition of this article conforms to the requirements of the amended International Code of Zoological Nomenclature, and hence the new names contained herein are available under that Code from the electronic edition of this article. This published work and the nomenclatural acts it contains have been registered in ZooBank, the online registration system for the ICZN. The ZooBank LSIDs (Life Science Identifiers) can be resolved and the associated information viewed through any standard web browser by appending the LSID to the prefix “http://zoobank.org/”. The LSID for this publication is: urn:lsid:zoobank.org:pub:8C96D835-EB8F-4664-9CA1- 99A0E2CDAA4B. The electronic edition of this work was published in a journal with an ISSN, and has been archived and is available from the following digital repositories: PubMed Central, LOCKSS.

### Phylogenetic Analysis

To determine the systematic positions of the Jehol frogs studied in this paper, we conducted a phylogenetic analysis of 25 taxa and 65 characters in PAUP 4.0b10 using the Branch-and-Bound search option. All characters were unordered and equally weighted, and ACCTRAN optimization was used to minimize the possibility of parallelisms. The resulting trees were rooted by using Caudata as an outgroup. The taxon-character matrix (see Table S1) was adapted from Wang, 2006 [13] with some amendments to the character descriptions and coding. We exclude the invalid taxa “*Callobatrachus*”, “*Mesophryne*”, and “*Yizhoubatrachus*” from the Jehol Biota of China, as well as the disarticulated material from Japan (the Tetori frog). The amended matrix also reflects our revised view, described below of the species-level taxonomy of *Liaobatrachus*. Character definitions mainly follow Gao and Wang, 2001 [10] and Wang, 2006 [13] with the following amendments:

**Character 3**: Medial contact between nasals: contact present, or nasals slightly separated medially (0); nasals widely separated medially (1); or nasals fused medially (2).

**Character 31**: Parahyoid: plate-shaped (circular or triangular plate) (0); V-shaped splint (1); absent (2).

**Character 50**: Shape of coracoid: medial end of coracoid very broadly expanded (0); medial end of coracoid moderately expanded, about as wide as distal end (1); medial end of coracoid slightly expanded (2).

## Systematic Paleontology

Amphibia Gray, 1825

Anura Fischer von Waldheim, 1813

Family incertae sedis

*Liaobatrachus* Ji and Ji, 1998

1998 *Liaobatrachus* Ji and Ji, p. 39, fig. 1

1999 *Callobatrachus* Wang and Gao, p. 637, fig. 1

2001 *Mesophryne* Gao and Wang, p. 461, figs 2–4

**Figure 3:**
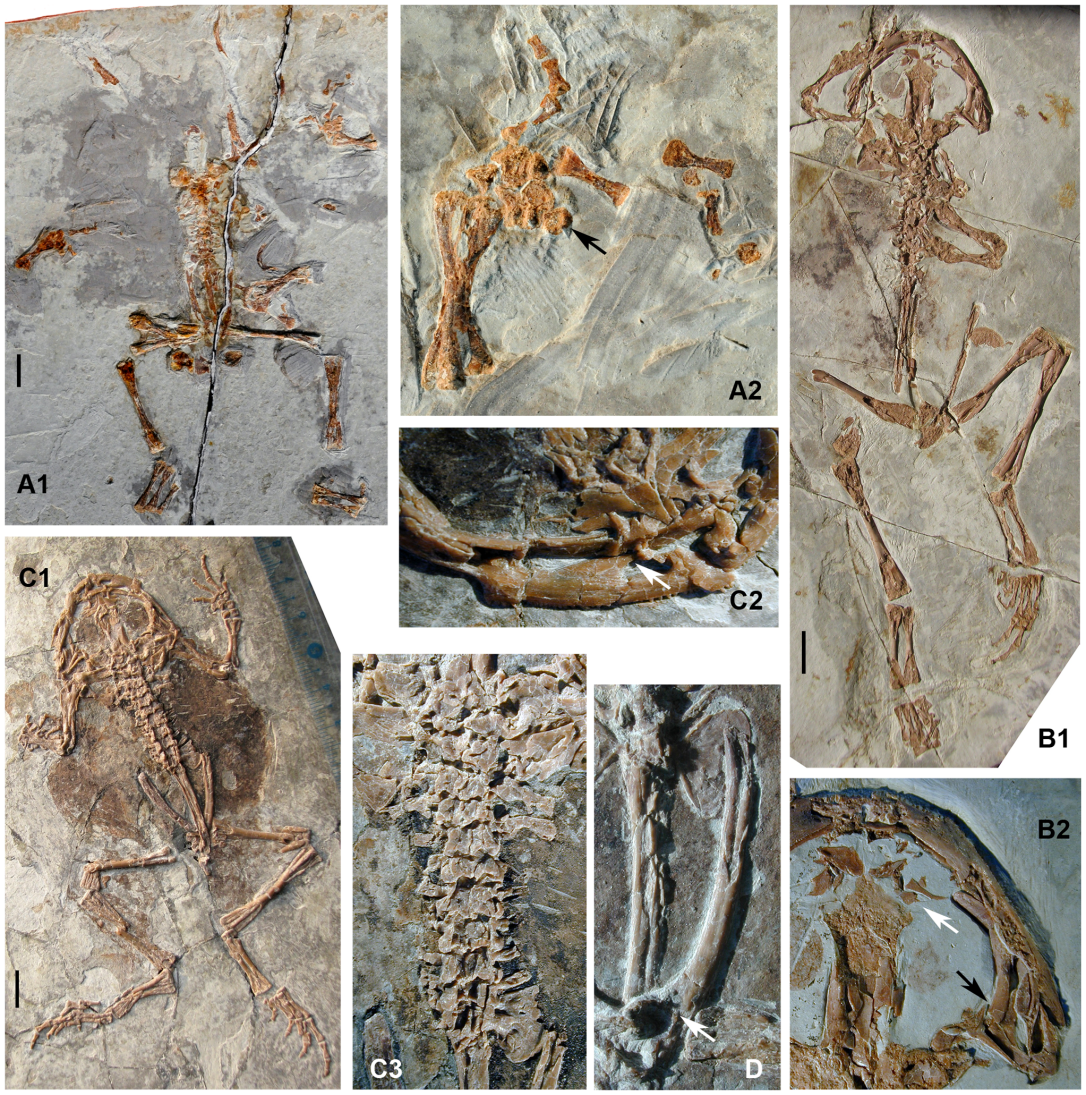
*Liaobatrachus grabaui* Ji and Ji, 1998. A1, skeleton of the holotype, GMV2126. A2, enlargement of the left radioulna, carpals and hand. The carpals are arranged in three rows: a proximal one containing the radiale and ulnare, a middle one containing distal carpal V and element Y, and a distal one containing distal carpals II–IV. There is one prepollex element situated medial to element Y (indicated by arrow). B1, skeleton of the referred specimen IVPP V11525. B2, enlargement of part of the skull. The long postchoanal process of the vomer is marked by a white arrow, and the short, free zygomatic ramus of the squamosal by a black arrow. C1, skeleton of the referred specimen MV 77. C2, enlargement of the premaxilla and the anterior end of the maxilla. The arrow marks the bifurcated anterior end of the maxilla. C3, enlargement of the vertebral column. There are 10 presacrals and the last one is partially fused with the sacrum, a condition that probably represents a developmental anomaly. D, ilium of the referred specimen CYH 004. The round acetabulum is marked by an arrow.

**Figure 4:**
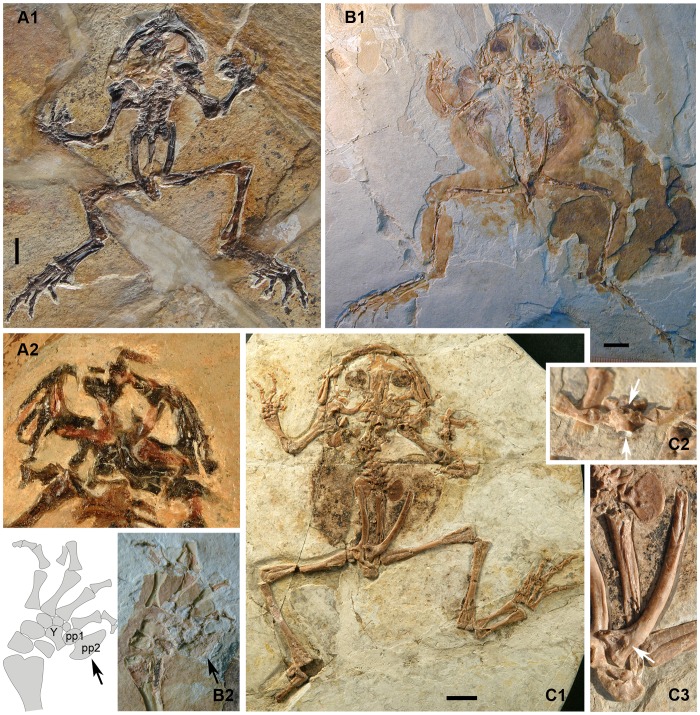
*Liaobatrachus beipiaoensis* (Gao and Wang, 2001) comb. nov. A1, skeleton of the holotype, LPM 0030. A2, skull of the holotype as preserved on the missing counterpart slab. B1, skeleton of the referred specimen DNM D2167. B2, photograph and line drawing of the hand of the specimen DNM D2167. The carpals are arranged in three rows and there are two prepollex elements (the larger, distally positioned one is marked by an arrow). C1, skeleton of the referred specimen IVPP V12717. C2, enlargement of presacral IV. The two arrows indicate the concave anterior and posterior surfaces of the amphicoelous centrum. C3, ilium of IVPP V12717. The acetabulum (marked by an arrow) is more or less rounded. Abbreviations: pp, prepollex; Y, element Y.

2004 *Dalianbatrachus* Gao and Liu, p. 2, fig. 1; pl. 1, fig. 1

2004 *Yizhoubatrachus* Gao and Chen, p. 762, figs 2–3

### 

#### Type species

*Liaobatrachus grabaui* Ji and Ji, 1998

#### Revised diagnosis

A crown-group anuran having the following unique combination of characteristics: medium body size (snout-vent length [SVL] 62∼94 mm, with location of vent in living animal considered to correspond to that of posterior end of ischium); skull wider than long; dermal roofing bones unsculptured; maxilla deep and bifurcated anteriorly; quadratojugal present; nasals with extensive midline contact; a large fontanelle between paired frontoparietals; squamosal with short zygomatic ramus that does not contact maxilla; vomer with dentigerous portion bearing 6–10 teeth arranged in single row and long postchoanal process (as long as, or longer than prechoanal process), which forms narrow angle with prechoanal process; parahyoid V-shaped; columella present; nine amphicoelous presacrals; atlas with type II cotyles (located ventral to the foramen magnum and adjacent to each other [17]); three pairs of recognizable ribs on presacrals II–IV (either unfused with corresponding transverse processes, or coalesced with transverse processes but with boundary recognizable in each case as thickened or unossified seam); sacral diapophyses broadly dilated and fan-like with convex lateral edges; sacro-urostylar articulation monocondylar; urostyle with one pair of transverse processes; scapula short and discoglossid-like whose anterior margin is straight and whose anteromedial margin is overlapped by distal end of clavicle; cleithrum not bifurcated medially; ilium with no dorsal protuberance or dorsal crest.

**Comparisons** (with leiopelmatids, discoglossids, and other Jurassic and Cretaceous anurans). In contrast to extant leiopelmatids [18]–[22], *Liaobatrachus* has nasals that contact one another medially (instead of lacking midline contact), a quadratojugal and columella (bones that absent in leiopelmatids), neural arches that are imbricated (instead of non-imbricated with the spinal canal partially exposed), sacral diapophyses that are fan-like (instead of rod-like), and a monocondylar sacro-urostylar articulation (instead of a non-condylar one). *Liaobatrachus* is similar to leiopelmatids in lacking dermal sculpture on the cranial roofing bones, having paired frontoparietals that bound a fontanelle, lacking a free palatine, and having nine amphicoelous presacral vertebrae, a cleithrum that is not distally bifurcated, and recognizable ribs present on presacral II–IV.

*Liaobatrachus* differs from discoglossids [23]–[26] in having a maxilla with a bifurcated anterior end (instead of an unbifurcated one), a cleithrum that is not distally bifurcated, nine amphicoelous presacral vertebrae (instead of eight opisthocoelous ones), a monocondylar sacro-urostylar articulation (bicondylar in most discoglossids, although monocondylar in *Barbourula*), and an ilium that lacks a dorsal protuberance and dorsal crest. *Liaobatrachus* and discoglossids share many characteristics, such as: free palatine absent, paired frontoparietals with fontanelle present, quadratojugal present, anteromedial margin of scapula overlapped by distal end of clavicle, second through fourth presacral vertebrae bear recognizable ribs, and rib on third presacral with uncinate process, sacral diapophyses dilated, one pair of transverse processes on urostyle.

*Liaobatrachus* differs from *Prosalirus* Shubin and Jenkins 1995 [27] from the Lower Jurassic of Arizona in having a simplified columella with an expanded footplate and round shaft (rather than bearing one ventral proximal head as in *Prosalirus*), a maxilla with a bifurcated anterior end (rather than unbifurcated), fan-like sacral diapophyses (instead of rod-like), a monocondylar sacro-urostylar articulation (instead of a non-condylar articulation), and a relatively long urostyle.

*Liaobatrachus* is distinguished from *Vieraella* Reig 1961 [28] from the Lower Jurassic of Argentina by being larger (SVL = 33 mm in *Vieraella*), and by having a fused prootic and exoccipital, a cultriform process of the parasphenoid that is long and reaches the level of the vomers (rather than relatively short as in *Vieraella*), fewer presacral vertebrae (10 in *Vieraella*) and fan-like sacral diapophyses (instead of rod-like). However, *Liaobatrachus* has a greater number of similarities with *Vieraella* than with *Prosalirus*. For example, both *Liaobatrachus* and *Vieraella* are characterized by extensive contact between the nasals, and by having a fontanelle between the frontoparietals.

*Liaobatrachus* is different from *Notobatrachus* Reig 1956 [28], [29] from the Middle to Upper Jurassic of Argentina, in being smaller (SVL: 120∼140 mm in *Notobatrachus*), lacking dermal sculpture on the cranial roofing bones, displaying contact between the nasals and between the frontoparietals, having a single well-ossified sphenethmoid (instead of a pair of ossification centers, sometimes called “orbitosphenoids”), having a fused prootic and exoccipital, having a V-shaped parahyoid (rather than crescent shaped), having fan-like sacral diapophyses (instead of rod-like), lacking discrete postsacral vertebrae (one discrete postsacral vertebra is present in most *Notobatrachus* specimens), and having a monocondylar sacro-urostylar articulation (rather than a non-condylar one). The two taxa are similar in having nasals with an extensive midline contact, paired frontoparietals surrounding a fontanelle, a vomer with a dentigerous portion, nine amphicoelous presacral vertebrae, imbricated neural arches, an atlas with type II cotyles, recognizable ribs, and an ilium without a dorsal protuberance or dorsal crest.

*Liaobatrachus* differs from *Eodiscoglossus* Villalta 1957 [30], [31] from the Middle and Upper Jurassic of Europe in having nasals with an extensive midline contact (midline contact absent in *Eodiscoglossus*), nine amphicoelous presacrals (instead of eight opisthocoelous ones), and an ilium without a dorsal protuberance or dorsal crest (both present on the ilium of *Eodiscoglossus*). *Liaobatrachus* is similar to *Eodiscoglossus* in lacking dermal sculpture on the cranial roofing bones, bearing a fontanelle between the paired frontoparietals, and having a low and long coronoid process on the angulosplenial, recognizable ribs only on presacrals II–IV, imbricated neural arches, fan-like sacral diapophyses, and one pair of transverse processes on the urostyle.

*Liaobatrachus* differs from *Wealdenbatrachus* Fey 1988 [32], a supposedly *Eodiscoglossus*-like taxon from the Lower Cretaceous of Spain, in at least two respects: the ilium lacks a dorsal crest (strong dorsal crest present in *Wealdenbatrachus*) and the sacral diapophyses are fan-like (rather than rod-like).

*Liaobatrachus* differs from *Gobiates* Špinar and Tatarinov 1986 [33], [34] and *Cretasalia* Gubin 1999 [34], [35] from the Upper Cretaceous of Central Asia in the following respects: (1) cranial dermal sculpture: in *Gobiates* pit-ridge sculpture is present on all dermal roofing cranial bones, dermal sculpture in *Cretasalia* is limited to the maxilla, and *Liaobatrachus* lacks dermal sculpture; (2) morphology of maxilla: in *Gobiates* this bone is not bifurcated anteriorly and has a small facial process, whereas in *Liaobatrachus* the facial process is proportionally larger and the anterior end is bifurcated into dorsal and ventral rami (unknown in *Cretasalia*); (3) contact between nasals: the nasals of *Gobiates* and *Cretasalia* are in contact only anteromedially, whereas *Liaobatrachus* has extensive midline contact; (4) squamoso-maxillary contact: in *Gobiates* and *Cretasalia* the zygomatic ramus of the squamosal is long and in contact with the maxilla, whereas in *Liaobatrachus* the squamosal and maxilla do not contact one another; (5) morphology of vomer: the vomer of *Gobiates* has no postchoanal process, whereas that of *Liaobatrachus* bears a long postchoanal process (unknown in *Cretasalia*); (6) cultriform process of parasphenoid: the cultriform process of *Gobiates* does not reach the level of vomers, whereas that of *Liaobatrachus* does (unknown in *Cretasalia*); (7) fusion of the prootic and exoccipital: in *Gobiates* and *Cretasalia* the two bones are separated by a suture, instead of fully fused as in *Liaobatrachus*. However, these three anurans also share many similarities. For example, the frontoparietals of all three bound a large fontanelle; the presacrals are amphicoelous; the neural arches are strongly imbricated; the urostyle has one pair of transverse processes; and the ilium lacks a dorsal crest.

*Liaobatrachus* also differs from *Cordicephalus* Nevo 1968 and *Thoraciliacus* Nevo 1968 [36]–[38] from the Lower Cretaceous of the Middle East in a number of respects. These include (1) vertebral number: seven or eight presacral vertebrae present in *Cordicephalus* and *Thoraciliacus*, compared to nine in *Liaobatrachus*; (2) vertebral structure: opisthocoelous with well developed transverse processes in *Cordicephalus*, but amphicoelous with short transverse processes in *Liaobatrachus* (unknown in *Thoraciliacus*); (3) vomerine teeth: absent in *Cordicephalus* (unknown in *Thoraciliacus*), but present and arranged in a single row in *Liaobatrachus*; (4) parasphenoid shape: lateral wings absent in *Cordicephalus* and *Thoraciliacus*, but present in *Liaobatrachus*; (5) frontoparietal morphology: broad and azygous in *Cordicephalus* and *Thoraciliacus*, but paired in *Liaobatrachus*; and (6) size: *Cordicephalus* and *Thoraciliacus* small-bodied (SVL: 30∼40 mm), but *Liaobatrachus* large-bodied (SVL: 62∼94 mm).

*Liaobatrachus* differs from *Aygroua* Jones, Evans and Sigogneau-Russell 2003 [39] from the Lower Cretaceous of Morocco with respect to iliac structure: the ilium of *Aygroua* has a strong dorsal crest, flared ventral acetabular rim, and prominent medial buttress, whereas the ilium of *Liaobatrachus* has none of these features.

#### Remarks

*Callobatrachus*, *Mesophryne*, *Dalianbatrachus* and *Yizhoubatrachus* are re-assigned to *Liaobatrachus* based on thorough examination of their holotypes and newly collected fossils from Sihetun, Qianyangou, and Hejiaxin localities. All of the latter fossils can also be referred to this genus as detailed below.

### *Liaobatrachus grabaui* Ji and Ji, 1998

Figure 3A∼D

1998 *Liaobatrachus grabaui* Ji and Ji, p. 39, fig. 1

1999 *Callobatrachus sanyanensis* Wang and Gao, p. 637, fig. 1

#### Holotype

GMV2126, incomplete skeleton in dorsal view, with vertebral column and pelvis articulated but skull and limbs disarticulated and displaced.

#### Type locality and horizon

Sihetun Locality; Jianshangou Bed, Yixian Formation, Barremian/Aptian (125±1 Ma) [40]–[42].

#### Referred specimens

IVPP V11525 (holotype of “*Callobatrachus sanyanensis*”) (Fig. 3B), MV 77 (Fig. 3C), CYH 004 (Fig. 3D).

#### Revised diagnosis

The type species of *Liaobatrachus* differs from other species referred to this genus in having the following unique combination of features: relatively short hind limbs, tibiofibula and femur subequal in length, maxilla with palatine process, and only one prepollex element present.

#### Description

CYH 004 (SVL = 78.6 mm), IVPP V11525 (SVL = 94 mm) and MV 77 (SVL = 83.5 mm) represent adult frogs. However, GMV2126 (SVL≈75 mm) probably represents a young adult, judging by the absence of ossified epiphyses on the long bones.

The skull is wider than long. No sculpture is present on the dermal roofing bones, maxilla, or squamosal (IVPP V11525, MV 77). The nasals are in extensive contact with each other medially (IVPP V11525, MV 77), and each nasal possesses an obvious rostral process which extends to the distal end of the alary process of the premaxilla (MV 77). The anterolateral margin of the nasal is moderately concave, and a low parachoanal process is present halfway along its length (IVPP V11525, MV 77). The paraorbital process is long and directed laterally, forming the anterior margin of the orbit together with the maxilla (CYH 004, MV 77). The paired frontoparietals are separated by a suture along the posterior portion (GMV2126), and anteriorly they bound a large fontanelle that extends for more than half the total length of the frontoparietals (GMV2126, IVPP V11525, MV 77). The orbital margin is straight, and a frontoparietal shelf is present (IVPP V11525). The structure of the alae of the parasphenoid is unknown due to poor preservation. The prootic and exoccipital are fused in adults (GMV2126, CYH 004, IVPP V11525, MV 77). The columella (IVPP V11525, MV 77) has a swollen footplate and bar-like shaft. The squamosal (IVPP V11525) is T-shaped, with three rami. The zygomatic ramus is short and does not contact the maxilla. The otic ramus articulates with the otic capsule, but does not contact the frontoparietal. The ventral ramus is the longest of the three, and extends ventrally to articulate with the quadratojugal.

The premaxilla (IVPP V11525, MV 77) bears about 24 tooth positions. The base of the alary process is perpendicular to the main body, but the distal end of the process is directed laterally. The development of the horizontal lamina is unknown due to the fact that the premaxillae are exposed only in dorsal view. The posterior process is absent (CYH 004). The maxilla bifurcates anteriorly into ventral and dorsal rami (GMV2126, IVPP V11525, MV 77), and the ventral ramus articulates with the premaxilla (MV 77). In life, the longer dorsal ramus likely had a ligamentous connection with the alary process of the premaxilla (MV 77). The facial process (GMV2126, MV 77) is prominent, and perpendicular to the main body of the maxilla. The disarticulated right maxilla of GMV2126, exposed in ventral view, reveals the presence of a palatine process and also shows that the zygomatico-maxillary process is less well developed than the facial process (GMV2126). The maxilla extends posteriorly to the level of the otic capsule (IVPP V11525, MV 77). The number of maxillary teeth is unknown, but exceeds 30. The quadrate is ossified and fused with the quadratojugal, completing the maxillary arch (CYH 004, IVPP V11525, MV 77).

The vomer bears a plate-like anterior portion located just posterior to the articulation between the premaxilla and maxilla (IVPP V11525, MV 77). The prominent prechoanal and postchoanal processes of the vomer are directed laterally and define a narrow angle (IVPP V11525, MV 77). The morphology of the ventral aspect of the vomer is unknown, as this bone is exposed dorsally in all specimens in which it is preserved. A short ossified portion of the nasal septum projects from the sphenethmoid (IVPP V11525, MV77). The parasphenoid is exposed only in IVPP V11525 and MV 77, in both cases through the frontoparietal fontanelle, and the important feature of the lateral portion cannot be observed in either specimen. The pterygoid is triradiate (CYH 004, GMV2126, IVPP V 11525, MV 77): the anterior ramus is the longest of the three and articulates with the posterior portion of the maxilla, the medial ramus partially overlaps the anterior surface of the otic capsule, and the posterior ramus articulates with the quadratojugal.

The lower jaw is composed of a mentomeckelian bone (MV 77), dentary and angulosplenial (CYH 004, IVPP V11525). The angulosplenial bears a low coronoid process. A notch is not present on the coronoid process. Only the posteromedial process of the hyoid plate can be observed (CYH 004, IVPP V11525).

The vertebral column generally consists of nine clearly amphicoelous presacral vertebrae (IVPP V11525), the sacral vertebra, and the urostyle. However, there are 10 presacral vertebrae in MV 77, and the last presacral vertebra of this specimen is partially fused to the sacral vertebra. The left transverse process of the tenth presacral and the left sacral diapophysis are distally fused to form an enlarged sacral diapophysis, but remain separated proximally. We regard this occurrence as a developmental anomaly, as is true of a similar morphological pattern that has previously been reported in extant anurans [43]. The neural arches are imbricated (GMV2126, IVPP V11525, MV 77), and bear low spines which probably do not extend posteriorly beyond the posterior margins of the neural arches (MV 77). Presacrals II–IV possess recognizable ribs that are either free from the transverse processes of the vertebrae (IVPP V11525, MV 77) or fused with them (GMV2126, MV 77). The ribs of presacral II are “hatchet-like”, each bearing an uncinate process at the distal end. The ribs of presacral III are longer than those associated with presacral II, and each bears an uncinate process located at the midlength. The ribs of presacral IV lack uncinate processes. The posterior presacrals of the vertebral column have transverse processes that are either nearly perpendicular to the body axis (GMV2126) or only slightly inclined anteriorly (CYH 004, IVPP V11525, MV 77). The sacral diapophyses are dilated and fan-like, with convex lateral margins (GMV2126, CYH 004, IVPP V11525, MV 77). The urostyle is long, and a pair of short transverse processes is present on the anterior part of this bone (GMV2126, IVPP V11525).

The clavicle is strongly curved (GMV2126, CYH 004, IVPP V11525), with a finger-like lateral end that partially overlaps the anterior surface of the pars acromialis of the scapula (CYH 004, IVPP V11525). The sulcus for the precoracoidal cartilage can be seen in IVPP V11525 and MV 77. The medial and lateral ends of the coracoid are nearly equal in width (IVPP 11525, MV 77). The short scapula has a straight or slightly concave anterior margin (GMV2126, CYH 004, IVPP V11525, MV 77), and a deep interglenoidal sinus between the pars acromialis and pars glenoidalis (GMV2126, IVPP V11525). The cleithrum has a nearly straight anterior margin (GMV2126, CYH 004, IVPP V11525, MV 77) and is not distally bifurcated (GMV2126, CYH 004, IVPP V11525).

The humerus bears a well developed ventral crest proximally (GMV2126, MV 77), and a deep cubital fossa (MV 77). The radial epicondyle is poorly developed whereas the ulnar one is moderately well developed. The medial and lateral crests are absent (MV 77), suggesting that the skeleton probably belongs to a female. The radioulna bears a well developed olecranon process (GMV2126, CYH 004, IVPP V11525, MV 77). The carpals are arranged in three rows: a proximal one containing the radiale and ulnare, a middle one containing distal carpal V and element Y, and a distal one containing distal carpals II–IV (GMV2126, CYH 004, MV 77) [44]. One large prepollex element is located medial to the second finger (GMV2126, CYH 004 MV 77) [45]. The phalangeal formula of the manus is 2-2-3-3 (IVPP V11525, MV 77).

The ilium does not have a dorsal crest or dorsal protuberance, and is slightly swollen where the acetabular region meets the shaft (GMV2126, IVPP V11525, MV 77). No further morphological details can be discerned, due to poor preservation. The ischium is kidney-shaped (CYH 004, IVPP V11525, MV 77), and the pubis is unossified.

The femur is sigmoid in shape, and its proximal portion bears a weak femoral crest (CYH 004, IVPP V11525, MV 77). The tibiofibula is slightly shorter than the femur and shows a nutrient foramen at the midlength (CYH 004, IVPP V11525, MV 77). The tibiale and fibulare are free from one another (GMV2126, IVPP V11525, MV 77). The distal tarsals are unossified and the phalangeal formula of the pes is 2-2-3-4-3 (IVPP V11525, MV 77).

#### Remarks

The holotype of *Liaobatrachus grabaui* (GMV2126) was first described in 1998. At that time *Liaobatrachus* was mistakenly assigned to the family Pelobatidae based on the supposed presence of several characteristics [8], including: frontoparietals fused together, procoelous presacrals, presacrals II–IV with long diapophyses (but no ribs), and fan-like sacral diapophyses. However, re-examination of GMV2126 reveals that this specimen has three pairs of recognizable ribs rather than long presacral transverse processes, and that the frontoparietals are paired. The type of the centrum in GMV2126 cannot be determined due to poor preservation of the specimen. Therefore, referral of *Liaobatrachus grabaui* to the Pelobatidae is not supported.

“*Callobatrachus sanyanensis*” (IVPP V11525) was the second named Jehol anuran, and this specimen is better preserved than the holotype (GMV2126) of *Liaobatrachus grabaui*. In previous studies [9], [10], [13], IVPP V11525 was assigned to the Discoglossidae based on the supposed presence of opisthocoelous presacral centra and a bicondylar sacro-urostylar articulation. However, more thorough preparation of the fossil and re-interpretation of its osteology suggest that the presacral centra are in fact amphicoelous and that the sacro-urostylar articulation cannot be referred to as bicondylar. Furthermore, this specimen differs from GMV2126 only in the degree of ossification of the olecranon process of the radioulna, a difference that is probably ontogenetic. Therefore, “*Callobatrachus sanyanensis*” is here considered to represent a junior synonym of *Liaobatrachus grabaui*.

### *Liaobatrachus beipiaoensis* (Gao and Wang, 2001) comb. nov

Figure 4A∼C

2001 *Mesophryne beipiaoensis* Gao and Wang, p. 461, figs 2–4

2004 *Dalianbatrachus mengi* Gao and Liu, p. 2, fig. 1, pl. 1, fig. 1

#### Holotype

LPM 0030, a nearly complete skeleton exposed on part and counterpart slabs, with the skull slightly distorted.

#### Type locality and horizon

Heitizigou locality; Jianshangou Bed, Yixian Formation, Barremian/Aptian (125±1 Ma).

#### Referred specimens

DNM D2166/7 (holotype of “*Dalianbatrachus mengi*”) (Fig. 4B), IVPP V12717 (Fig. 4C).

#### Revised diagnosis

This species differs from other *Liaobatrachus* species in having the following unique combination of characteristics: relatively long hind limbs, femur slightly longer than tibiofibula, ilium with subcircular acetabulum, and two prepollex elements present.

#### Description

LPM 0030 (SVL = 69 mm), DNM D2166/7 (SVL = 73.5 mm), and IVPP V12717 (SVL≈83 mm) are all adults.

As in the type species of *Liaobatrachus*, the skull is relatively wide and lacks sculpture on the cranial bones (LPM 0030, DNM D2167). Medial contact between the nasals is extensive (LPM 0030, DNM D2167, IVPP V12717), and the parachoanal process is present but relatively weakly developed (LPM 0030, IVPP V12717). The paraorbital process extends laterally, but fails to reach the maxilla and thus leaves the anterior orbital margin incomplete (LPM 0030, DNM D2167). The paired frontoparietals are in contact with each other posteriorly, but bound a large fontanelle anteriorly (IVPP V12717). The lateral edges of these bones are straight, and each bears a shelf (DNM D2167, IVPP V12717). Behind the orbit the frontoparietals expand laterally but do not form prominent alae (IVPP V12717). Specimen IVPP V12717 shows that the prootic and exoccipital are fused, and that the columella is composed of a swollen footplate and bar-like shaft. The T-shaped squamosal (IVPP V12717) has a short zygomatic ramus that does not contact the maxilla; the ventral ramus is the longest of the three, and extends ventrally to articulate with the ossified quadrate.

There are about 23 tooth positions on the dental lamina of the premaxilla (LPM 0030). The anterior portion of the maxilla is dorsoventrally bifurcated (LPM 0030), and the ventral ramus articulates with the premaxilla. The dorsal ramus is longer than the ventral one, and is directed anterodorsally (LPM 0030). The maxilla extends posteriorly to the level of the otic capsule (IVPP V12717). The number of tooth positions on the maxilla cannot be determined. The quadratojugal, together with the premaxilla and maxilla, completes the maxillary arch (IVPP V12717).

The vomer has a plate-like anterior portion (DNM D2166), and prominent prechoanal and postchoanal processes which are directed laterally and define a narrow angle between them (LPM 0030, DNM D2166). The dentigerous area is not preserved. The cultriform process of the parasphenoid has a rounded tip (IVPP V12717) and reaches the level of the vomer (DNM D2166). The parasphenoid alae are narrow (their anteroposterior length being less than 1/3 of the distance between their lateral ends); the lateral and posterior margins of the alae are obscured by postcranial bones in available specimens. Thus, whether the parasphenoid bears a posterolateral notch and posterior process is unknown. The pterygoid is triradiate (LPM 0030, DNM D2167, IVPP V12717), and articulates with the maxilla, otic capsule, and quadratojugal.

The lower jaw is formed by the coalesced mentomeckelian bone and dentary (LPM 0030, DNM D2167), and by the angulosplenial. The angulosplenial (LPM 0030, DNM D2167) has a low coronoid process, but it is not possible to assess whether notches are present on the anterior and posterior margins of the process.

There are nine presacral vertebrae. The centra of the presacrals are clearly amphicoelous (DNM D2167, IVPP V12717). The neural arches are imbricated and bear low spines (IVPP V12717). The atlantal cotyles (IVPP V12717) are ventrally located and close to each other, and can be recognized as type II of Lynch (1971). Recognizable ribs are associated with presacrals II–IV. These may be free (LPM 0030, IVPP V12717) or fused to the transverse processes (DNM D2167). The ribs of presacral II bear uncinate processes at their tips (LPM 0030, DNM D2167, IVPP V12717), whereas those of presacral III have uncinate processes at the midlength (LPM 0030, IVPP V12717). The transverse processes of the posterior presacrals are generally anteriorly inclined (DNM D2167, IVPP V12717), but in some cases extend straight laterally (LPM 0030). As in the type species, the sacral diapophyses are fan-like (LPM 0030, DNM D2167, IVPP V12717). The sacro-urostylar articulation is clearly monocondylar (IVPP V12717). The urostyle bears a pair of short transverse processes (LPM 0030) and a spinal nerve foramen, but no dorsal crest is present (IVPP V12717).

The curved clavicle (DNM D2167) has a finger-like lateral end that partially overlaps the anterior surface of the pars acromialis of the scapula (DNM D2167, IVPP V12717). The medial end of the bar-like coracoid is slightly wider than the lateral end (CYH 004, IVPP V12717). The scapula is short, with a slightly concave anterior margin (LPM 0030, DNM D2167, IVPP V12717) and an interglenoidal sinus separating the pars acromialis and pars glenoidalis (DNM D2167). The cleithrum is not distally bifurcated (LPM 0030, IVPP V12717), and the anterior margin of this bone is nearly straight (IVPP V12717).

The humerus has a well developed ventral crest (LPM 0030, DNM D2167, IVPP V12717). In specimen IVPP V12717 this bone lacks radial and ulnar epicondyles in addition to medial and lateral crests, probably reflecting sexual dimorphism and indicating a female individual. The cubital fossa is deep (IVPP V12717). The radioulna has a well developed olecranon process (LPM 0030, IVPP V12717). The carpus is configured as in the type species (DNM D2166, IVPP V12717). Two prepollex elements, of which the distal one is the larger, are located medial to the second finger (LPM 0030, DNM D2167). The phalangeal formula of the manus is 2-2-3-3 (DNM D2167, IVPP V12717).

The ilium has neither a dorsal crest nor a dorsal protuberance (IVPP V12717). The pars ascendens is moderately developed (IVPP V12717) but the pars descendens (IVPP V12717) is barely discernible. The ischia are fused (LPM 0030, IVPP V12717) and the pubes are unossified.

The proximal portion of the sigmoid femur bears a crest (IVPP V12717). The tibiofibula, which is nearly as long as the femur, shows a nutrient foramen (IVPP V12717). The tibiale and fibulare are free from one another (LPM 0030, DNM D2166/7, IVPP V12717). The distal tarsals are not ossified, and the phalangeal formula of the pes is 2-2-3-4-3 (LPM 0030, DNM D2167, IVPP V12717).

#### Remarks

LPM 0030 was catalogued as IVPP V11721 when it was made the holotype of the new putative “*Mesophryne beipiaoensis*” [10], but was subsequently returned to the municipal government of Beipiao and catalogued in the Liaoning Paleontology Museum under its present specimen number. Only the part slab (published as IVPP V11721A, now LPM 0030), which preserves less of the skull than the counterpart, is currently available. The counterpart slab was probably lost after being returned to Liaoning. Contrary to the original description of the specimen [10], the carpus of LPM 0030 does not contain an intermedium and the presacral centra cannot be determined to be procoelous. The specimen has the general characteristics of the genus *Liaobatrachus*, including a bifurcated maxilla, a nasal and vomer of the appropriate shape, recognizable ribs on presacrals II–IV, fan-like sacral diapophyses, and transverse processes on the urostyle, and is reassigned to *Liaobatrachus* in this paper to form the new combination *L*. *beipiaoensis*.

Different catalogue numbers (DNM D2166, DNM D2167) have been given to the part and counterpart of the holotype of “*Dalianbatrachus mengi*”, even though both components belong to the same individual [11]. DNM D2166/7 has paired frontoparietals and an amphicoelous presacral centrum, and displays virtually no differences from LPM 0030. Thus we regard “*Dalianbatrachus mengi*” as a junior synonym of *Liaobatrachus beipiaoensis*.

### *Liaobatrachus macilentus* (Gao and Chen, 2004) comb. nov

Figure 5A, B

**Figure 5:**
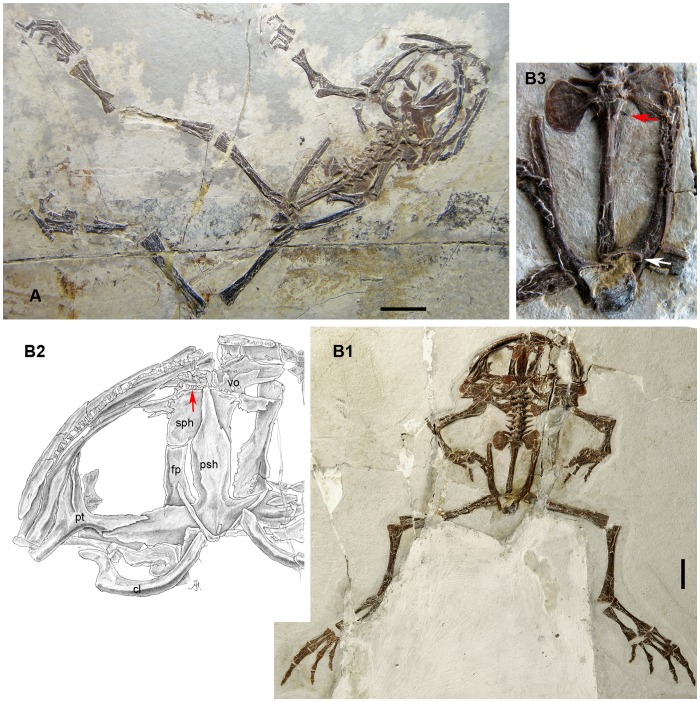
*Liaobatrachus macilentus* (Gao and Chen, 2004) comb. **nov.** **** A, skeleton of the holotype, ZMNH M8621. B1, skeleton of the referred specimen IVPP V12510. B2, line drawing of the skull, with an arrow indicating the vomerine tooth row. B3, enlargement of the pelvis. The acetabulum is pointed anteriorly, and marked by a white arrow. The urostyle possesses one pair of transverse processes, which are indicated by a red arrow. Abbreviations: cl, clavicle; fp, frontoparietal; psh, parasphenoid; pt, pterygoid; sph, sphenethmoid; vo, vomer.

2004 *Yizhoubatrachus macilentus* Gao and Chen, p. 762, figs 2–3

#### Holotype

ZMNH M8621, nearly complete skeleton with some cranial bones slightly displaced, and metacarpals and phalanges of left forelimb preserved as imprint.

#### Type locality and horizon

Hejiaxin locality; Dawangzhangzi Bed, Yixian Formation, Aptian (122 Ma) [2].

#### Referred specimens

IVPP V12510 (Fig. 5B), IVPP V12541.

#### Revised diagnosis

This species differs from other species of *Liaobatrachus* in having the following unique combination of features: femur slightly longer than tibiofibula, maxilla bearing a palatine process, cultriform process of parasphenoid ending in slender needle-like tip (tapering abruptly at a point located about 1/3 of the skull length posterior to the rostrum), anterior acetabular margin of ilium pointed anteriorly, and two prepollex elements present.

#### Description

IVPP V12510 (SVL = 62 mm) is an adult, but is not fully grown, and ZMNH M8621 (SVL = 56 mm) is a juvenile.

As in the type species of *Liaobatrachus*, the skull is relatively wide. The nasal has a long paraorbital process which is directed laterally and forms the orbital margin together with the maxilla (ZMNH M8621). The frontoparietals are paired and border a large anterior fontanelle (IVPP V12510). The orbital margin is straight and a frontoparietal shelf is present (ZMNH M8621, IVPP V12510). In ZMNH M8621, the medial margin of the anterior portion of the frontoparietal is thickened. The posterior portions of the frontoparietals are sutured together, and extend slightly laterally behind the orbits (IVPP V12510). The otic capsules are nearly completely overlapped in ventral view by the alae of the parasphenoid (IVPP V12510), and a faint suture can be recognized between the prootic and exoccipital. The columella (ZMNH M8621, IVPP V12510) has a swollen footplate and bar-like shaft, and the T-shaped squamosal (IVPP V12510) has a short zygomatic ramus that does not contact the maxilla.

There are 18∼20 tooth positions on the dental lamina of the premaxilla (IVPP V12510, ZMNH M8621). The alary process extends dorsally, but the distal end of the process is laterally inclined. The anterior portion of the maxilla is deep, and probably bifurcated (ZMNH M8621). The facial process is prominent and nearly perpendicular to the main body of the maxilla. The palatine process is well developed, and positioned anterior to the facial process (ZMNH M8621). The horizontal lamina widens posteriorly (ZMNH M8621), and thus probably possesses a pterygoid process. The posterior end of the maxilla reaches the level of the otic capsule, and is obtuse and rounded (IVPP V12510). The maxilla bears 41∼47 tooth positions (ZMNH M8621, IVPP V12510), and the tooth row extends beyond the level of the pterygoid process. The quadratojugal is present, and is fused with the ossified quadrate (IVPP V12510).

The vomer has prominent prechoanal and postchoanal processes, which diverge laterally at a narrow angle from one another (ZMNH 8621, IVPP V12510). A dentigerous area with about 6 tooth positions arranged in a single row is present close to the midline, medial to the choana. The sphenethmoid has a prominent anterior process, representing the ossified part of the nasal septum. The parasphenoid has a cultriform process that reaches the level of the vomer. The process is slightly constricted at its base, and widens gradually up to its midpoint before tapering to a slender and needle-like tip (ZMNH M8621, IVPP V12510). The nature of the alae and posterior margin is unknown. The pterygoid (ZMNH M8621, IVPP V12510) is triradiate: the anterior ramus is the longest of the three and extends anteriorly to articulate with the maxilla, whereas the medial ramus contacts the otic capsule.

The lower jaw is formed mainly by the coalesced mentomeckelian bone and dentary (ZMNH M8621, IVPP V12510), and the angulosplenial. The angulosplenial has a low but distinct coronoid process, with a smooth posterior margin and an anterior margin that is probably interrupted by a notch (ZMNH M8621). A V-shaped parahyoid and bar-like posteromedial process of the hyoid plate are preserved (ZMNH M8621, IVPP V12510).

The axial column is comprised of nine presacral vertebrae, the sacral vertebra, and the urostyle. The atlas and presacral II are fused in IVPP V12510, but this is probably an individual anomaly. The centra of the presacrals are clearly amphicoelous (ZMNH M8621, IVPP V12510) and the atlantal cotyles (IVPP V12510) are of type II of Lynch (1971). Recognizable ribs are present, and free from the corresponding transverse processes, on presacrals II–IV (IVPP V12510). Those of presacral III bear uncinate processes directed posterolaterally from their middle portions. The transverse processes of the posterior presacrals are inclined somewhat anteriorly (ZMNH M8621, IVPP V12510), and the sacral diapophyses are obviously fan-like (ZMNH M8621, IVPP V12510). The urostyle is longer than the presacral vertebral column and bears a pair of short transverse processes (IVPP V12510).

The clavicle is strongly arched, with a lateral end that partially overlaps the anterior surface of the pars acromialis of the scapula (IVPP V12510). The coracoid is bar-like and swollen at both ends, but the medial end is slightly wider than the lateral one (IVPP V12510). The scapula is short, with a straight anterior margin (ZMNH M8621, IVPP V12510) and a deep interglenoidal sinus between the pars acromialis and pars glenoidalis (ZMNH M8621). The cleithrum is not distally bifurcated, and its anterior margin is straight (ZMNH M8621, IVPP V12510).

The humerus bears a ventral crest (IVPP V12510). In specimen ZMNH M8621 the humeral condyle is not ossified, suggesting that the individual is a juvenile. The radial epicondyle is barely distinct, and the ulnar one is only moderately well developed (ZMNH M8621). The depth of the cubital fossa is uncertain due to poor preservation. The carpals (radiale, ulnare, element Y, and distal carpals II–V) are arranged in three rows (IVPP V12510) as in *Liaobatrachus grabaui*. Two prepollex elements are present, the distal one being larger than the proximal (IVPP V12510). In the juvenile ZMNH M8621, the carpals are not ossified. The phalangeal formula of the manus is 2-2-3-3 (IVPP V12510).

The ilium does not have a dorsal crest or dorsal protuberance, and is somewhat rugose at the point of transition between the acetabular portion and the shaft (ZMNH M8621, IVPP V12510). The anterior margin of the acetabulum (ZMNH M8621, IVPP V12510) is pointed anteriorly rather than rounded, and the anterior and ventral margins both rise to form crests. The pars ascendens is moderately well developed, but the pars descendens is nearly lacking (ZMNH M8621, IVPP V12510). The ischia are fused, and kidney-like (IVPP V12510). The pubes were cartilaginous.

The femur is slightly longer than the tibiofibula (ZMNH M8621, IVPP V12510), and the latter bone bears a nutrient foramen at its midpoint (IVPP V12510). The tibiale and fibulare are not fused together (ZMNH M8621, IVPP V12510). The distal tarsals were cartilaginous. The phalangeal formula of the pes is 2-2-3-4-3 (IVPP V12510).

#### Remarks

Wang (2006) pointed out that the holotype of “*Yizhoubatrachus macilentus*” (ZMNH M8621) actually represents a medium-sized subadult frog (SVL: 56∼62 mm) rather than a large frog as reported in the original paper (SVL = 115 mm according to Gao and Chen, 2004, page 766), so the lack of an ossified mesopodium in both the fore- and hind limbs is likely a non-diagnostic subadult feature. This conclusion is further supported by discovery of a larger specimen (IVPP V12510) from the same locality and closely resembling ZMNH M8621, which does have ossified carpals and probably represents an adult. Moreover, whether the parasphenoid of ZMNH M8621 has a posterior process is uncertain, because the posterior margin is obscured in the available specimens. The presacral centra are amphicoelous, not opisthocoelous as previously reported. This degree of conformity with the diagnosis of *Liaobatrachus* indicates that “*Yizhoubatrachus*” is a junior synonym of *Liaobatrachus*.

### *Liaobatrachus zhaoi* sp. nov

urn:lsid:zoobank.org:act:1D921AAE-054E-4768-A953-E312572AB6C0

**Figure 6:**
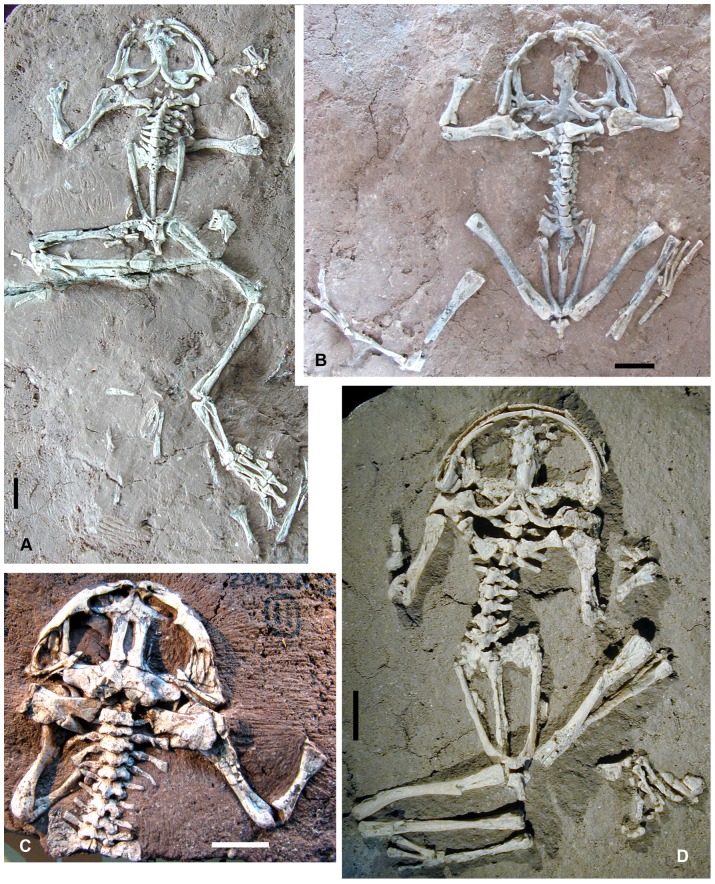
*Liaobatrachus zhaoi* sp. nov. A, the holotype, IVPP V14979.1. B, the paratype specimen IVPP V14979.2. C, the paratype specimen IVPP V13239. D, the paratype specimen IVPP V14203.

**Figure 7:**
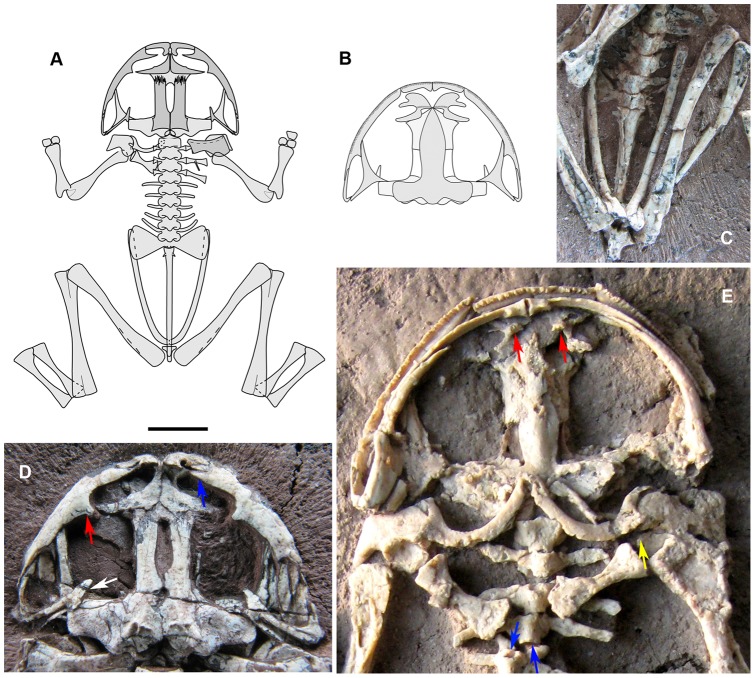
*Liaobatrachus zhaoi* sp. nov. A, restoration of the skeleton of *L*. *zhaoi* in dorsal view. As shown, the left pectoral girdle includes the clavicle, scapula, and coracoid when the cleithrum and the ribs of presacrals II–IV have been removed. B, restoration of the skull of *L*. *zhaoi* in ventral view. C, pelvic girdle of the referred specimen IVPP V13245. D, skull of the paratype specimen IVPP V13239 in dorsal view. The blue arrow marks the bifurcated anterior end of the maxilla, the red arrow marks the prominent facial process of the maxilla, and the white arrow marks the free zygomatic ramus of the squamosal. E, enlargement of the anterior portion of the paratype specimen IVPP V14203 in ventral view. The red arrow points to the dentigerous portion of the vomer, which bears one row of teeth. The yellow arrow marks the interglenoidal sinus between the pars acromialis and pars glenoidalis. The blue arrows show the concave surfaces of the amphicoelous presacral centra.

Figure 6A∼D, Figure 7A∼E

2012 *Liaobatrachus* Ji and Ji, Roček et al., p. 1287, figs 1, 2

#### Etymology

The species name honors Professor Ermi Zhao, a prominent Chinese herpetologist.

#### Holotype

IVPP V14979.1, a nearly complete, three-dimensionally preserved skeleton exposed in ventral aspect, the largest and most complete of four specimens preserved on a single block of silty sandstone.

#### Paratypes

IVPP V14979.2, a nearly complete, three-dimensionally preserved skeleton in ventral aspect. IVPP V14203, a nearly complete, three-dimensionally preserved skeleton in ventral aspect, with only the distal parts of the forelimbs missing. IVPP V13239, a partial, three-dimensionally preserved skeleton in dorsal aspect, retaining the skull, the presacral section of the vertebral column and parts of the forelimbs but lacking the pelvis and hind limbs.

#### Type locality and horizon

Qianyangou locality; Lujiatun Bed, Yixian Formation, Barremian (slightly older than 125 Ma) [2], [42], [46].

#### Referred specimens

IVPP V13236, IVPP V13245 (Fig 7C), IVPP V13380, IVPP V14269, IVPP V14270, IVPP V14979.3, IVPP V14979.4, probably IVPP V13238, IVPP V13379, and IVPP V14608. All are from the type locality and surrounding area (Lujiatun Village).

#### Diagnosis

This new species of *Liaobatrachus* differs from other members of the genus in having the following unique combination of characteristics: relatively long hind limbs, tibiofibula nearly as long as femur, parasphenoid with cultriform process that tapers gradually beginning at the midpoint, maxilla without palatine process, ilium with round acetabulum, and pubis ossified in fully grown adults.

#### Description

(based on type specimens) IVPP V14979.1 (SVL = 73.8 mm), IVPP V13239, and IVPP V14203 (SVL = 80.5 mm) are fully grown adults, whereas IVPP V14979.2 (SVL = 73 mm) is an early adult.

The skull is wider than long. No sculpture is present on the dermal roofing bones, maxilla, or squamosal (IVPP V 13239, IVPP V 14203). The nasals contact one another extensively along their medial margins (IVPP V13239) and each possesses an obvious rostral process, which extends as far as to the level of the alary process of the premaxilla (IVPP V14979.1, IVPP V13239). The anterolateral margin is moderately concave (IVPP V14979.1, IVPP V13239), and a low parachoanal process is present at the midpoint of the margin (IVPP V13239). The paraorbital process (IVPP V13239) is long and laterally directed, forming the anterior margin of the orbit together with the maxilla. The paired frontoparietals are sutured together posteriorly, and their anterior portions border a large fontanelle that is more than half as long as the frontoparietal (IVPP V13239). The orbital margin is straight, and a frontoparietal shelf is present. The posterior portion of the frontoparietal extends laterally but is not developed as wing-like process, and the lateral process, paraoccipital process, posterior process and posterolateral canthus can all be seen (IVPP V13239). The prootic and exoccipital are completely fused with each other on both sides of the skull (IVPP V13239). The supracondyloid crest extends from the posterolateral canthus of the frontoparietal to the torus terminalis. The two occipital condyles, located near each other, are mainly ventral to the foramen magnum. A columella (IVPP V13239) is present. The squamosal (IVPP V13239) is T-shaped. The short zygomatic ramus does not contact the maxilla, and is directed anteriorly and medially. The otic plate articulates with the otic capsule, but is not in contact with the frontoparietal.

The premaxilla bears about 20 tooth positions (IVPP V14203). The alary process (IVPP V13239) is short. The basal portion of the alary process is perpendicular to the main body of the premaxilla, and the distal portion is directed laterally. The horizontal lamina of the premaxilla (IVPP V14203) has a poorly developed palatine process but no posterior process. The maxilla bifurcates into ventral and dorsal rami anteriorly, and only the ventral ramus articulates with the premaxilla (IVPP V14203). The dorsal ramus is the longer of the two, and probably had a ligamentous connection with the alary process of the premaxilla in the living animal (IVPP V13239). The maxilla bears at least 48 tooth positions (IVPP V14979.2). The facial process (IVPP V13239, IVPP V14203) is prominent, and perpendicular to the main body of the maxilla. The palatine process is absent (IVPP V14979.2), and the zygomatico-maxillar process is small (IVPP V13239). The maxilla extends posteriorly to the level of the otic capsule (IVPP V13239, IVPP V14979.2). The quadrate is ossified and fused with the quadratojugal, which articulates with the posterior end of the maxilla (IVPP V13239, IVPP V14203, IVPP V14979.2).

The vomer has a plate-like anterior portion (IVPP V14203), and the prominent prechoanal and postchoanal processes of this bone are directed laterally and define a narrow angle (IVPP V13239, IVPP V14203, IVPP V14979.1). The dentigerous portion is located medial to the choana, and bears 10 teeth arranged in a single row. The sphenethmoid is long in fully grown adults (IVPP V14203, IVPP 14979.1) but poorly developed in young adults, in which the nasal septum is cartilaginous (IVPP V14979.2). The cultriform process of the parasphenoid, whose base is slightly constricted, extends anteriorly to the level of the vomer (IVPP V13380). The cultriform process is widest at the midpoint, and tapers gradually as it continues anteriorly. The alae are long and narrow, their anteroposterior width being less than 1/3 of the distance between their lateral ends. There is no notch (IVPP V14979.1) in the posterolateral margin of the ala, but a distinct posterior process (IVPP V14979.2) is present in the middle of the posterior margin. The anterior ramus of the pterygoid articulates with the pterygoid process of the maxilla (IVPP V14203, IVPP V149797.1, IVPP V14979.2). The medial ramus of the pterygoid (IVPP V13239) partially overlaps the anterior surface of the otic capsule, and the posterior ramus (IVPP V14979.1, IVPP V14979.2) contacts the ventral ramus of the squamosal.

The lower jaw is comprised of a mentomeckelian bone (IVPP V14203, IVPP V 14979.2), a dentary (IVPP V14203, IVPP V14979.1, IVPP V14979.2), and an angulosplenial that bears a low but distinct coronoid process lacking a notch (IVPP V14203). A V-shaped parahyoid bone is present (IVPP V14979.2), and the posteromedial process of the hyoid apparatus is visible (IVPP V14203).

The nine presacral vertebrae are clearly amphicoelous (IVPP V14203, IVPP V14979.1, IVPP V14979.2). The atlas and presacral II (IVPP V13380), or the last presacral vertebra and the sacral vertebra (IVPP V 13236), are sometimes fused. This condition is here regarded as an individual anomaly, and has been reported previously in extant frogs [20], [43], [47]. The neural arches are imbricated and bear low spines (IVPP V13239). The atlantal cotyles (IVPP V14203), which are recognizably of type II (of Lynch, 1971), are close to each other and approximately ventral in position. Presacrals II–IV bear recognizable ribs, which are either free from the transverse processes of the vertebrae (IVPP V14203, IVPP V14979.2) or coalesced with them (IVPP V13239). An uncinate process is present at the tip of the rib of presacral II, making the rib “hatchet-like”, and an uncinate process also occurs at the middle of the rib of presacral III (IVPP V13239). Recognizable ribs are occasionally present on presacral V, in cases where the area of fusion between the rib and the transverse process is swollen (IVPP V13236, IVPP V13239). The transverse processes of presacrals V–VII are perpendicular to the body axis, whereas those of the last two presacrals are inclined anteriorly (IVPP V13239, IVPP V14203, IVPP V14979.1, IVPP V14979.2). The sacral diapophyses are fan-like (IVPP V14203, IVPP V14979.1). The sacro-urostylar articulation is monocondylar (IVPP V14979.1, IVPP V14979.2). The urostyle has a pair of short transverse processes (IVPP V14203, IVPP V14979.1), and bears only a poorly developed dorsal crest (IVPP V13236).

The clavicles are strongly curved, and meet in the midline (IVPP V14979.1). The lateral, finger-like end of each clavicle overlaps the anterior surface of the pars acromialis of the scapula (IVPP V13239, IVPP V14203). The medial and lateral ends of the bar-like coracoid are nearly equal in width (IVPP V14203, IVPP V14979.2). The short scapula has a straight anterior margin (IVPP V13239, IVPP V14203, IVPP V14979.1) and a deep interglenoidal sinus between the pars acromialis and pars glenoidalis (IVPP V14203). The cleithrum has a straight anterior margin and does not bifurcate distally (IVPP V13239).

The humerus has a well developed ventral crest on its proximal portion (IVPP V 14979.1, IVPP V14979.2), and the cubital fossa is deep (IVPP V13239, IVPP V14979.1). The humeral condyle may be either ossified (IVPP V13239, IVPP V14979.1) or unossified (IVPP V14203), due to developmental variation. The radial epicondyle is less well developed than the ulnar one. Medial and lateral crests are not present (IVPP V13245, IVPP V14203). The radioulna bears a well developed olecranon process (IVPP V14979.1, IVPP V14979.2). Ossified carpals are present, but the arrangement of the carpals and the phalangeal formula of the manus are both unknown due to incomplete preservation.

The ilium does not bear a dorsal crest or a dorsal protuberance (IVPP V13236). The pars ascendens is moderately well developed (IVPP V14203). The pars descendens is not laterally expanded, but combines with the ventral rim of the acetabulum to form a small platform (IVPP V14203, IVPP V14979.1, IVPP V14979.2). In a fully grown adult (IVPP V14979.1), the ilium, ischium, and ossified pubis all contribute to a confluent ventral acetabular rim. In young adults (IVPP V14203, IVPP V14979.2) the pubis was still cartilaginous, as indicated by the presence of an empty gap where the pubis would be expected to occur. The fused ischia are T-shaped in dorsal view (IVPP V14979.1, IVPP V14979.2).

The femur is sigmoid in shape and bears a crest on the proximal section (IVPP V14203, IVPP V14979.1, IVPP V14979.2). The tibiofibula is nearly as long as the femur (IVPP V14979.1). The tibiale and fibulare are fused at their proximal and distal ends in fully grown adults (IVPP V14979.1), but unfused in younger adults (IVPP V14979.2). Similarly, the distal tarsals are ossified in fully grown adults (IVPP V 14979.1), but not in younger ones (IVPP V14979.2). The phalangeal formula of the pes is unknown due to poor preservation.

### Anura genus and species indet

#### Material

IVPP V13235, part and counterpart of a partial skeleton.

#### Horizon and locality

Jiufotang Formation (Aptian, 120.3±0.7 Ma) [48]; Xierhuqiao Locality.

#### Description

Each vertebra has an unfused neural arch and centrum, and the long bones lack epicondyles. These features indicate that the individual is surely a juvenile (for detailed description see Wang et al., 2007 [16]).

The frontoparietals are paired, and their anterior portions border a large fontanelle whose anteroposterior length exceeds 1/2 the length of the frontoparietals. The squamosal is T-shaped, with short zygomatic ramus that does not contact the maxilla. The maxilla is not bifurcated anteriorly and lacks a facial process. The sacral diapophyses are rod-like and only slightly expanded at their ends, rather than being fan-like as in *Liaobatrachus*. The sacro-urostylar articulation is bicondylar, also in contrast to the condition in *Liaobatrachus*. The urostyle bears a pair of short, posteriorly directed transverse processes. The tibiofibula is considerably longer than the femur. The tibiale and fibulare are not fused together, and the distal tarsals are not ossified.

A ternary diagram was used to analyze the hind limb proportions of anurans and two outgroup taxa [16], and this frog from the Jiufotang Formation was well separated on the diagram from a cluster of Jehol anurans from the underlying Yixian Formation. In fact, the Jiufotang frog fell amongst extant hylids, leptodactylids, and discoglossids. The precise phylogenetic position of this small frog remains uncertain, but the specimen differs from *Liaobatrachus* in having a greater ratio of tibiofibula length to femur length and having undilated sacral diapophyses. The Jiufotang frog is probably not referable to *Liaobatrachus,* and the possibility that it represents a neobatrachian cannot be ruled out.

## Discussion

All fossil anuran specimens that belong to the Jehol Biota come from the Yixian Formation, with the exception of one specimen IVPP V13235 that comes from the Jiufotang Formation. It is clear that, with respect to osteological characters, the Yixian frogs are homogeneous and should be referred to the single genus *Liaobatrachus*.

Within *Liaobatrachus* we distinguish four species according to body proportions, the shape of the cultriform process of the parasphenoid, the shape of the acetabulum, and the number of prepollex elements. The relative length of the hind limb (femur, tibiofibula, and fibulare) is greater in *L*. *beipiaoensis* and *L*. *zhaoi* than in *L*. *grabaui* (see Table S2). However, in *L*. *beipiaoensis* the femur is slightly longer than the tibiofibula, as opposed to nearly the same length as the tibiofibula in *L*. *zhaoi*. The acetabulum is triangular in *L*. *macilentus* but more circular in other species of *Liaobatrachus*. The cultriform process of the parasphenoid is anteriorly needle-like in *L*. *macilentus*, but tapers gradually anteriorly in *L*. *zhaoi* and comes to a rounded anterior tip in *L*. *beipiaoensis*. *Liaobatrachu*s *grabaui* possesses only one prepollex element whereas *L*. *beipiaoensis* and *L*. *macilentus* have two. Other differences among the specimens exist, but can be explained as intraspecific variations or developmental anomalies. The intraspecific differences are mainly ontogenetic variations in the degree of ossification. For example, the young adult of *L*. *macilentus* has unossified carpals and a poorly developed radioulna that lacks a prominent olecranon; the fully grown adult of *L*. *zhaoi* has distinct epicondyles at the ends of the long bones, an ossified pubis and distal tarsals, and a proximally and distally fused tibiale and fibulare. Further examples of inferred developmental anomalies include the presence, in some specimens, of an additional presacral (presacral X) in *L*. *grabaui*, of the fusion between presacrals I and II in *L*. *macilentus*, and of the fusion between the last presacral and the sacral vertebra in *L*. *grabaui* and *L*. *zhaoi*.

*Liaobatrachus* exhibits several derived character states not seen in *Prosalirus*, *Notobatrachus* and *Vieraella* from the Jurassic deposits of the Americas. These include a reduced number of presacrals, a complete urostyle, and a monocondylar sacro-urostylar articulation as opposed to a non-condylar joint between the sacrum and urostyle. However, *Liaobatrachus* does not appear to be as derived as the European Jurassic-Cretaceous *Eodiscoglossus*, as the presacrals of *Liaobatrachus* are amphicoelous and also more numerous. *Gobiates* and *Cretasalia* from the Upper Cretaceous of Central Asia resemble *Liaobatrachus* more closely than American and European taxa in possessing an ilium that lacks a dorsal crest and having at least some contact between the nasals. However, *Liaobatrachus* differs from these Central Asian taxa in having a greater degree of contact between the nasals and in lacking both a squamosal-maxillary contact and dermal sculpture on the bones of the skull roof. *Liaobatrachus* does not resemble the Early Cretaceous frogs known from the Middle East or North Africa, which are pipoids.

**Figure 8:**
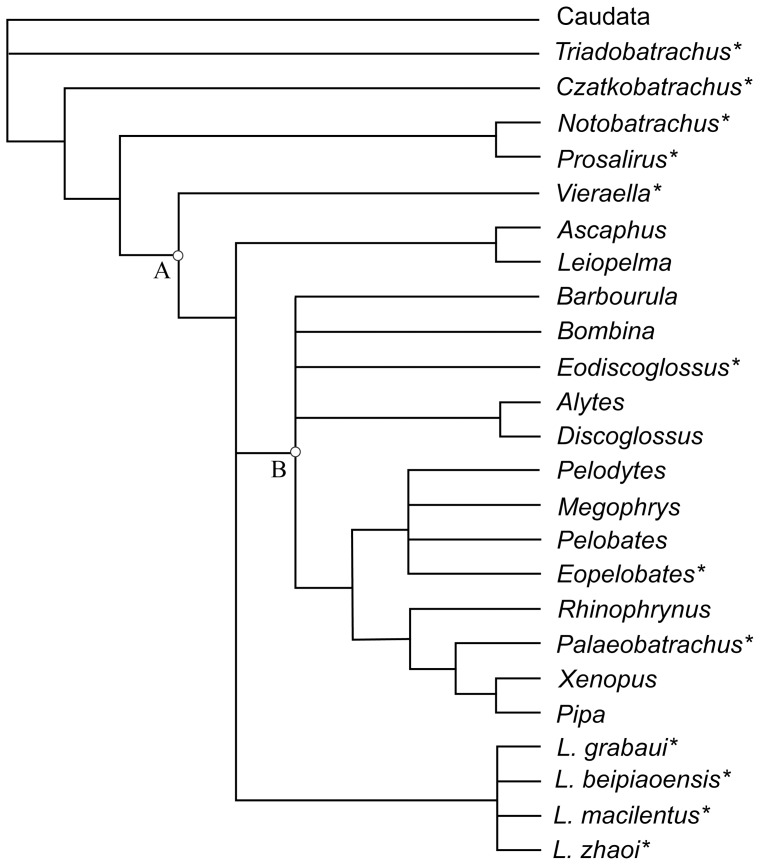
Phylogenetic placement of *Liaobatrachus* among archaeobatrachian frogs. The tree is the strict consensus of 661 most parsimonious trees (TL = 186, CI = 0.5753, RI = 0.6985). The data matrix used in the analysis is in Table S1. Major phylogenetic groups shown on the tree include (A) Anura and (B) Lalagobatrachia. * indicates fossil taxa.

Among extant anurans, *Liaobatrachus* resembles leiopelmatids in some features and discoglossids in others. As in discoglossids, the anterior margin of the pars acromialis of the scapula is overlapped by the distal end of the clavicle, and the sacral diapophyses are expanded. As in leiopelmatids, nine presacral vertebrae are present and the distal end of the cleithrum is unbifurcated. However, the combination of amphicoelous centra and a monocondylar sacro-urostylar articulation suggests that *Liaobatrachus* is likely more derived than leiopelmatids. In our phylogenetic analysis (Fig. 8), *Liaobatrachus* is clustered together with leiopelmatids and the Lalagobatrachia (of Frost et al., 2006 [49]). *Liaobatrachus* is more deeply nested than most previous analyses have indicated [10], [12], [13], [50].

The frog from the Jiufotang Formation of Aptian age is about 5 million years younger than the known specimens of *Liaobatrachus*, and differs from them in having rod-like sacral diapophyses and larger ratio of tibiofibula to femur. This frog provides evidence of the presence of another anuran genus in the Jehol Biota, but we prefer not to erect a new taxon on the basis of a single juvenile specimen.

## Conclusion

Most known Jehol anurans can be referred to the genus *Liaobatrachus* (Table 2) on the basis of osteological characters. The five previously named taxa, *Liaobatrachus grabaui* Ji and Ji 1998, *Callobatrachus sanyanensis* Wang and Gao 1999, *Mesophryne beipiaoensis* Gao and Wang 2001, *Dalianbatrachus mengi* Gao and Liu 2004 and *Yizhoubatrachus macilentus* Gao and Chen 2004, are consolidated into three species of *Liaobatrachus*: *L*. *grabaui*, *L*. *beipiaoensis* comb. nov. and *L*. *macilentus* comb. nov.. A new species, *Liaobatrachus zhaoi* sp. nov., is established based on a dozen three-dimensionally preserved specimens from the Lujiatun Bed of the Yixian Formation. Nevertheless, the known taxonomic diversity of the Jehol anurans is significantly less than previously believed. Comparisons with fossil and extant anurans, and a phylogenetic analysis, suggest that *Liaobatrachus* is a member of the anuran crown-group.

**Table 2:** Original names, holotypes, and revised names of anuran taxa from the Jehol Formation.

Previous name	Holotype	Age	Reference	Revised designation
‘Jiufotang frog’ (IVPP V13235)	N/A	120 Ma	Wang et al., 2007	Unnamed advanced frog
*Yizhoubatrachus macilentus*	ZMNH M8621	123 Ma	Gao and Chen, 2004	*Liaobatrachus macilentus*
*Liaobatrachus grabaui*	GMV 2126	125 Ma	Ji and Ji, 1998	*L*. *grabaui* *
*Callobatrachus sanyanensis*	IVPP V11525	125 Ma	Wang and Gao, 1999	*L*. *grabaui*
*Mesophryne beipiaoensis*	LPM 0030	125 Ma	Gao and Wang, 2001	*L*. *beipiaoensis*
*Dalianbatrachus mengi*	DNM D2166/7	125 Ma	Gao and Liu, 2004	*L*. *beipiaoensis*
[New taxon]	IVPP V14979.1	128 Ma	Roček et al. 2012;present paper	*L*. *zhaoi*

* indicates type species; N/A, not applicable.

## Supporting Information

Table S1 **Taxon-character data matrix used in our phylogenetic analysis.** Character description is as in Gao and Wang (2001) and Wang (2006) with amendments mentioned in the Material and Method section. Letters in bold indicate the character states that are rescored in this paper. The letters in the matrix represent the states shown below: A, (0&1); B, (1&2); C, (0 or 1); D, (0&2); E, (1 or 2).
(DOC)

Table S2 **Morphometric data of Jehol anurans.** Abbreviation: SVL, snout-vent length; PsC, presacral column; LS, length of skull; LtS, width of skull; H, humerus; Ru, radioulna; F, femur; Tf, tibiofibula; Fb, fibulare; I, ilium; U, Urostyle; Mt4, metatarsal IV.
(DOC)
