# Correction: Anurans from the Lower Cretaceous Jehol Group of Western Liaoning, China

**DOI:** 10.1371/annotation/bcdc57a0-1377-43a7-8336-7796533013c3

**Published:** 2013-12-16

**Authors:** Liping Dong, Zbyněk Roček, Yuan Wang, Marc E H. Jones

There is an error regarding the placement of 'A' in Figure 8. Please see the corrected Figure 8 here: http://plosone.org/corrections/pone.0069723.g008.cn.tif

