# Supplementary material for: Anurans from the Lower Cretaceous Jehol Group of Western Liaoning, China
Source: PLoS One. 2013 Jul 26;8(7):e69723. doi: 10.1371/journal.pone.0069723 (PMC3724893; doi:10.1371/journal.pone.0069723)
Supplement: Table S1 — Taxon-character data matrix used in our phylogenetic analysis. Character description is as in Gao and Wang (2001) and Wang (2006) with amendments mentioned in the Material and Method section. Letters in bold indicate the character states that are rescored in this paper. The letters in the matrix represent the states shown below: A, (0&1); B, (1&2); C, (0 or 1); D, (0&2); E, (1 or 2). (DOC) [file pone.0069723.s001.doc]

**Table S1** **Taxon-character data matrix used in our phylogenetic analysis.**

|  | 10 | 20 | 30 | 40 | 50 | 60 | 65 |
| --- | --- | --- | --- | --- | --- | --- | --- |
| Caudata | 000A000000 | 0000000000 | 000000--AA | -0000000?0 | ?000-0--0- | -00000-?00 | 00000 |
| *Triadobatrachus* | 000??10000 | 0????00000 | 01??0?0000 | 00?10?0000 | 00000000?? | 0100010000 | ????? |
| *Czatkobatrachus* | ?????????? | ?????????? | ?????????? | ??????0?0? | ?10??????? | ?110?1?0?? | ????? |
| *Prosalirus* | ?1???1??01 | ??1??????? | ?1????0?0? | ?0????0?0? | ?10E??10?0 | ??01?1?110 | ????0 |
| *Ascaphus* | 0110110210 | 1001111100 | 10-01011**0**0 | 010120012**2** | **0**11200111**1** | 0111110?11 | 01010 |
| *Leiopelma* | 0110110210 | 200**0**111100 | 101210111**0** | 0101200122 | 11**0**2001000 | 01**1**1110111 | 01110 |
| *Alytes* | 0100110210 | 2110120100 | 1112111110 | 1001301102 | 1112101111 | 1211111111 | 01100 |
| *Barbourula* | 010001**1**010 | 2100120100 | 1112111110 | **2**001301002 | 1122201111 | 1211110111 | 01100 |
| *Bombina* | 0100010**2**10 | 21**AA**130100 | 1112111110 | **D**001301002 | 112220111**2** | 1211110111 | 01100 |
| *Discoglossus* | 010**1**010**2**10 | 2110120110 | 1112111110 | **2**001301002 | 1112101111 | 1211111111 | 01100 |
| *Eodiscoglossus* | 010001011? | 1??0?20100 | 1????????? | ?0?13010?2 | 112210?1?1 | 1?1111?111 | ?1??0 |
| *Notobatrachus* | 00000101**1**1 | 1010011100 | 1001100001 | 00012?0021 | 0101001000 | 0101010110 | ????0 |
| *Vieraella* | **01**0?0101**1**0 | 1?**0**01??100 | 11111?010? | ??011?00?2 | 0?0????0?0 | 0?01?1??1? | ????? |
| *Pelobates* | 000001**1-**11 | 2110110111 | 1112011111 | 2001302004 | 112221110**1** | 0?11111111 | 12201 |
| *Eopelobates* | 0000010011 | ??????0111 | 11???11111 | ?0?13?20?4 | 112221?10**2** | ??11?10111 | 0???? |
| *Megophrys* | 0100010011 | 2?101?0101 | 11??11111? | 2001302004 | 1122111100 | 0?11111111 | 12201 |
| *Pelodytes* | 0**1**1**A**110**D**1? | 2?10110100 | 1112011110 | 1001312004 | 112220110**A** | ??11111112 | 01201 |
| *Pipa* | **A**100111**-**10 | 2201011300 | 12??102111 | 201B311003 | 1122321010 | 1?11110011 | 11201 |
| *Xenopus* | A120011**-**10 | 2301011300 | 12??102110 | 2011301023 | 112232112**2** | 1?11110011 | 11211 |
| *Rhinophrynus* | 1100011**-**11 | 2100110200 | 111?102102 | 0112301004 | 112212111**1** | 2211111111 | 11201 |
| *Palaeobatrachus* | 1100011**-**10 | 210001C100 | 111?102100 | **1**001312023 | 0112121110 | 1111A1?111 | 11??1 |
| *Liaobatrachus grabaui* | 01000102?1 | 2?00110100 | 1110101??1 | ?0012000C2 | 0122?01111 | 0111110110 | ?0??? |
| *Liaobatrachus beipiaoensis* | 0100010211 | 2?00?10100 | 11121011?1 | ?001200012 | 0122200111 | 0111110110 | ?0??? |
| *Liaobatrachus macilentus* | 010?010211 | 2?00110100 | 11121011?1 | 1001200012 | 0122201111 | 0111110110 | ?0??? |
| *Liaobatrachus zhaoi* | 0100010211 | 2?00110100 | 1112101111 | 1001200012 | 0122201111 | 0111110110 | ????? |

Character description is as in Gao and Wang (2001) and Wang (2006) with amendments mentioned in the Material and Method section. Letters in bold indicate the character states that are rescored in this paper. The letters in the matrix represent the states shown below: A, (0&1); B, (1&2); C, (0 or 1); D, (0&2); E, (1 or 2).
