# Supplementary material for: Anurans from the Lower Cretaceous Jehol Group of Western Liaoning, China
Source: PLoS One. 2013 Jul 26;8(7):e69723. doi: 10.1371/journal.pone.0069723 (PMC3724893; doi:10.1371/journal.pone.0069723)
Supplement: Table S2 — Morphometric data of Jehol anurans. Abbreviation: SVL, snout-vent length; PsC, presacral column; LS, length of skull; LtS, width of skull; H, humerus; Ru, radioulna; F, femur; Tf, tibiofibula; Fb, fibulare; I, ilium; U, Urostyle; Mt4, metatarsal IV. (DOC) [file pone.0069723.s002.doc]

**Table S2** **Morphometric data of Jehol anurans.**

|  | *L*. *grabaui* | | | | | *L*. *beipiaoensis* | | | *L*. *macilentus* | | *L*. *zhaoi* | | | | | | | | | | |  |
| --- | --- | --- | --- | --- | --- | --- | --- | --- | --- | --- | --- | --- | --- | --- | --- | --- | --- | --- | --- | --- | --- | --- |
| (mm) | GMV2126 | IVPP V11525 | MV 77 | CYH004 | IIICYGYB-002 | LPM0030 | DNM D2166(7) | IVPP V12717 | ZMNH M8621 | IVPP V12510 | I VPP V13236 | IVPP V13239 | IVPP V13245 | IVPP V13380 | IVPP V14203 | IVPP V14269 | IVPP V14270 | IVPP V14797.1 | IVPP V14797.2 | IVPP V13238 | IVPP V14608 | IVPP V13235 |
| SVL | ? | 94.0* | 83.5 | 78.6 | 87.5* | 69.0 | 73.5* | 83.0* | 56.1 | 62.0 | 70.5 | ? | 69.2 | ? | 80.5 | 67.0* | 65.0* | 73.8 | 73.0 | ? | ? | 38.0* |
| PsC | 22.0 | 28.4 | 27.5 | 23.0 | ? | 19.0 | 19.0 | 24.0* | 16.5* | 17.5 | 20.0* | 23.0 | ? | ? | 28.5* | 23.0* | 21.5 | 21.6* | 23.6 | ? | ? | ? |
| LS | ? | 28.5 | 25.0* | 22.5* | ? | ? | ? | ? | ? | 18.0 | 23.0 | 23.5 | ? | 22.5 | 23.0 | ? | 21.0 | ? | 22.5 | 22.5 | ? | ? |
| LtS | ? | 34.5 | 36.0 | 36 | ? | ? | ? | ? | ? | ? | ? | 35.5 | 30.0 | 32.5 | 35.0 | ? | 27.0 | 33.0 | 32.0 | 32.5 | ? | ? |
| H | ? | 20.9 | 20.0* | 22.7 | 22.7 | 23.5 | 19.0 | 19.6 | 10.9 | 18.8 | 27.8 | ? | 21.5 | ? | 20.0 | 19.0 | ? | 26.0? | 17.8 | ? | ? | ? |
| Ru | 13.5 | 13.6 | 13.0 | 15.0 | 17.3 | 14.1 | 13.0 | 14.0 | 7.7 | 12.2 | ? | 13.2 | 15.5 | ? | ? | ? | ? | 18.4 | 12.0 | ? | ? | ? |
| F | 29.0 | 34.2 | 30.5* | 34.4 | 34.6 | 31.1 | 33.5* | 34.5 | 22.8 | 30.0 | 29.7 | ? | 32.0 | ? | 33.0* | ? | 27.2 | 35.0 | 30.0 | 34.5 | 32.0 | 16.5 |
| Tf | 29.0 | 35.0 | 31.7 | 34.4 | 35.4 | 29.2 | 30.0 | 32.8 | 20.4 | 26.8 | 29.7 | ? | 32.0 | ? | 34.0 | ? | ? | 35.0 | 30.0* | 34.5 | 32.0 | 20.0 |
| Fb | 17.0 | 20.1 | 19.2 | 19.0 | 23.1 | 16.3 | 17.0 | 19.1 | 12.3 | 15.7 | 17.5 | ? | 16.5 | ? | ? | ? | ? | 21. 2 | ? | ? | ? | 10.2 |
| I | ? | 31.8 | ? | 26.9 | ? | ? | 27.1 | 30.0 | 17.5 | 23.3 | 27.5 | ? | 27.0 | ? | 27.5 | ? | ? | 28.5 | ? | ? | ? | 11.1 |
| U | ? | 27.8 | 25.7 | ? | ? | ? | ? | 32.0 | 18.2 | ? | 24.0 | ? | ? | ? | ? | ? | ? | ? | ? | 26.4 | ? | 9.1 |
| Mt4 | ? | 10.9 | 11.0 | 11.8 | 11.5 | 11.0 | 10.0 | 10.5 | 7.4 | 9.5 | ? | ? | ? | ? | ? | ? | ? | 12.7 | ? | ? | ? | 5.0 |
| PsC/SVL | ? | 0.30 | 0.33 | 0.29 | ? | 0.28 | 0.26 | 0.29 | 0.29 | 0.28 | 0.28 | ? | ? | ? | 0.35 | ? | ? | 0.29 | 0.32 | ? | ? | ? |
| LtS/LS | ? | 0.82 | 0.69 | 0.63 | ? | ? | ? | ? | ? | ? | ? | 0.66 | ? | 0.69 | 0.66 | ? | 0.77 | ? | 0.70 | 0.69 | ? | ? |
| U/SVL | ? | 0.30 | 0.31 | ? | ? | ? | ? | 0.39 | 0.28 | ? | 0.34 | ? | ? | ? | ? | ? | ? | ? | ? | ? | ? | 0.24 |
| (F+Tf+Fb)/SVL | ? | 0.95 | 0.98 | 1.12 | 1.06 | 1.16 | 1.10 | 1.04 | 0.99 | 1.17 | 1.09 | ? | 1.16 | ? | ? | ? | ? | 1.24? | ? | ? | ? | 1.25 |
| (F+Tf)/SVL | ? | 0.74 | 0.75 | 0.88 | 0.80 | 0.91 | 0.84 | 0.81 | 0.77 | 0.91 | 0.84 | ? | 0.93 | ? | 0.83 | ? | ? | 0.95 | 0.82 | ? | ? | 0.96 |
| Tf/F | 1.00 | 1.02 | 1.04 | 1.00 | 1.02 | 0.93 | 0.90 | 0.95 | 0.90 | 0.89 | 1.00 | ? | 1.00 | ? | 1.03 | ? | ? | 1.00 | 1.00 | 1.00 | 1.00 | 1.21 |
| Fb/F | 0.59 | 0.59 | 0.63 | 0.55 | ? | 0.52 | 0.51 | 0.55 | 0.54 | 0.52 | 0.59 | ? | 0.52 | ? | ? | ? | ? | ? | ? | ? | ? | 0.61 |
| Mt4/F | ? | 0.32 | 0.36 | 0.34 | 0.33 | 0.35 | 0.30 | 0.30 | 0.32 | 0.32 | ? | ? | ? | ? | ? | ? | ? | 0.36 | ? | ? | ? | 0.30 |

Abbreviation: SVL, snout-vent length; PsC, presacral column; LS, length of skull; LtS, width of skull; H, humerus; Ru, radioulna; F, femur; Tf, tibiofibula; Fb, fibulare; I, ilium; U, Urostyle; Mt4, metatarsal IV.
